# Acoustic Wave-Powered Durable Icephobic Duplex Coating Design with Superior Deicing Performance

**DOI:** 10.1021/acsami.5c19758

**Published:** 2026-01-30

**Authors:** Jaime del Moral, Luke Haworth, Laura Montes, Juan R. Sánchez-Valencia, Angel Barranco, Victor J. Rico, Triana Czermak, Francisco Carreño, Paloma García-Gallego, Julio Mora, Carmen López-Santos, Andreas Winkler, Ana Borrás, Agustín R. González-Elipe, Yongqing Fu

**Affiliations:** † Nanotechnology on Surfaces and Plasma Lab. Materials Science Institute of Seville, 16379 Consejo Superior de Investigaciones Científicas (CSIC - Univ. Sevilla), Americo Vespucio 49, 41092 Sevilla, Spain; ‡ Faculty of Engineering and Environment, 5995 Northumbria University, Newcastle upon Tyne, NE1 8ST, U.K.; § 54456 National Institute for Aerospace Technology (INTA), Ctra. Ajalvir km. 4, Torrejón de Ardoz, 28850, Spain; ∥ Departamento de Física Aplicada, Departamento de Física Aplicada I, 16778 Universidad de Sevilla, C/Virgen de Africa 7, Seville, 41011, Spain; ⊥ 28394 IFW Dresden, SAWLab Saxony, Helmholtzstr. 20, 01069 Dresden, Germany

**Keywords:** surface acoustic waves, deicing, anti-icing, Teflon-like, ZnO, diamond-like coating

## Abstract

Piezoelectric thin film-based surface acoustic wave (SAW) deicing technology has recently emerged as an attractive and energy-efficient alternative with direct applications across multiple industrial sectors. However, the generation of SAWs on piezoelectric thin films, such as ZnO, faces diverse challenges, including its low long-term stability and variable wetting properties upon exposure to UV radiation and other environmental hazards. To overcome these challenges, we propose a bilayer coating design that integrates a diamond-like carbon (DLC) thin film with an atop CF_*x*_ layer (DLC-CF_*x*_). This design is intended to serve as both an anti-icing and a protective coating for ZnO SAW devices built on aluminum substrates, which are specifically selected for critical ice-exposed applications in the aeronautics or wind turbine industries. We demonstrate that, unlike the implementation of single fluorinated polymer layers, such as commercial CYTOP, the DLC-CF_*x*_ hydrophobic duplex coating effectively protects the ZnO surfaces while maintaining optimal SAW transmission and wave propagation and reducing the fluorine content. The SAW-induced deicing on these devices is achieved through a highly effective mechanism involving the interfacial ice melting, followed by a rapid ice sliding detachment for both small ice droplets and large ice aggregates. Experiments at laboratory scale and in an icing wind tunnel facility reveal that deicing involves SAW activation of the interface between the ice and the DLC-CF_*x*_ bilayer, as well as an effective thermal contribution resulting from the rapid heat transmission through the aluminum substrate. Our studies demonstrate that the highly conformal deposition of DLC-CF_*x*_ through a room temperature plasma-assisted method ensures reliability and long-term stability of thin-film-based acoustic wave devices in harsh outdoor conditions.

## Graphical abstract

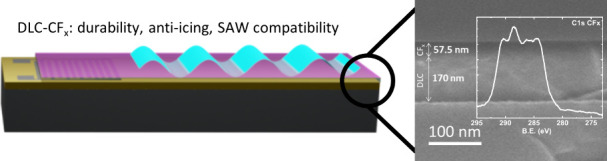

## 1 Introduction

Icing protection, including ice monitoring, deicing, and anti-icing, is critical for various industrial and energy sectors. Several passive and active strategies have been proposed to achieve highly efficient deicing while minimizing environmental impact, mainly including chemical, mechanical, electrothermal, photothermal, and ultrasonic approaches.^1–6^ Recently, the on-substrate acoustic wave (AW) generation has emerged as a versatile deicing method by inducing nanoscale mechanical vibrations on the AW activated surfaces.^7,8^ For example, thickness shear mode (TSM) waves,^9^ surface acoustic waves (SAWs),^10–12^ or Lamb waves,^13,14^ have been utilized for deicing and anti-icing on a variety of substrates, including aluminum,^10,11^ glass,^7^ fused silica,^12^ or piezoelectric ceramics.^9,12–14^ AWs have also been explored for detecting ice accretion (i.e., for ice sensing or ice thickness monitoring),^7,9,13,15–17^ which opens up the possibility for the smart operation or automation of integrated ice detection and removal systems.^9,13^ In this context, surface engineering of acoustic wave devices, including surface treatments and functional coatings compatible with acoustic wave transmission, represents an additional and advanced approach to manufacturing multifunctional systems that integrate both active deicing and passive anti-icing protection,^9,10,18^ while preserving the surface stability of the piezoelectric layers exposed to environmental conditions.

Piezoelectric films such as AlN, PZT, or ZnO have been deposited on a variety of substrates for the fabrication of SAW devices.^19,20^ Among them, ZnO has the merits of a relatively easy deposition and processing, a low film stress, and a relatively high electromechanical coupling coefficient if compared with AlN.^7,20,21^ Due to these features, ZnO thin film-based SAW devices for ice removal or prevention of ice accretion have been recently exploited for implementation on technically relevant substrates, such as aluminum,^10,11^ glass,^7^ and fused silica.^12^ However, a key issue is that harsh environmental conditions, such as those existing during icing, rain hazards, or prolonged exposure to the atmosphere, may affect the surface properties of ZnO and impair its stability and long-term outdoor usage. As is well-known, ZnO is easily etched away by acid or alkaline solutions, and its surface properties can be altered by prolonged exposure to humidity and moisture.^22^ Moreover, ZnO is a metal oxide semiconductor with a band gap of ∼3.2 eV, i.e., it can be readily excited by the UV light of the solar spectrum.^23^ When exposed to UV irradiation in air, the ZnO films undergo photochemical and photocatalytic surface reactions,^24,25^ which generate lattice defects and vacancies, induce the formation of intermediate reactive species on the surface, and lead to drastic changes in wettability. For instance, as-prepared ZnO thin films deposited by magnetron sputtering or plasma-enhanced chemical vapor deposition exhibit a hydrophilic character, but become less hydrophilic or even hydrophobic upon dark storage. This effect is attributed to contamination by airborne hydrocarbons. Additionally, when these aged ZnO thin films are exposed to sunlight or UV illumination, their surfaces quickly become highly hydrophilic or superhydrophilic, a process that slowly reverses in dark conditions.^26–28^ These uncontrollable changes in wettability in natural environments lead to poor reproducibility of the wetting and anti-icing properties of ZnO. Moreover, the mechanical robustness and wear resistance of ZnO films are highly dependent on the deposition technique and surface termination. Methods such as plasma-enhanced chemical vapor deposition (PECVD)^29^ and magnetron sputtering yield dense,^12,30^ homogeneous films with improved structural integrity and reliable conditioning of their interface with substrates. However, the application of protective coatings, while essential for mitigating surface wear, can significantly dampen acoustic wave transmission, particularly in surface acoustic wave devices, due to added mass loading and altered boundary conditions.^31^ To address these key issues, we report in this article on a new surface engineering strategy for achieving efficient acoustic wave transmission in ZnO-SAW devices fabricated on aluminum substrates, while providing stable deicing and anti-icing surface capabilities. Although grafted perfluoromolecules and fluoropolymer layers^32–36^ have been previously exploited as hydrophobic surfaces, herein, we go a step forward. We implement a multifunctional bilayer modification of SAW devices using diamond-like carbon (DLC) and a fluorinated polymer of CF_*x*_ (DLC-CF_*x*_), prepared by scalable and low-impact plasma-enhanced chemical vapor deposition. Structures and functionality of the coating are illustrated in Scheme 1. The bilayer coating offers the following advantages: (i) enhances the reliability and durability of the devices under harsh environmental conditions; (ii) ensures stable wetting and improved anti-icing performance; (iii) facilitates effective transmission of the SAW from the piezoelectric film to the device exposed surface; and (iv) enables efficient deicing, taking advantage of a lower ice adhesion strength due to the hydrophobic nature and low surface energy of the CF_*x*_ coating.

**Scheme 1:**
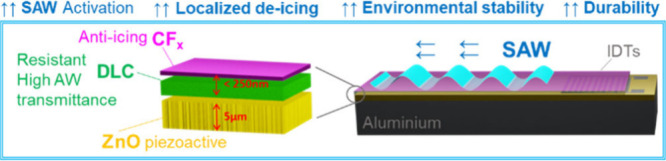
Scope of the Article and Schematic of the Device Layout for Al\ZnO\DLC-CF_*x*_ Systems

The proposed new design consists of a DLC-CF_*x*_ duplex coating prepared by plasma-assisted deposition in one-reactor configuration.^43,44^ This approach avoids exposing the samples to air between the deposition of each layer. The wear-resistant DLC layer is known for its high hardness, low friction, chemical inertness, proper adhesion to ZnO, and efficient acoustic transmission capability.^37^ On the other hand, the plasma-deposited CF_*x*_ overlayer provides good passive anti-icing behavior even for thicknesses of 100 nm or below. Moreover, from a manufacturing point of view, this bilayer configuration prevents direct contact of a fluorine-containing plasma with the ZnO substrate, thereby circumventing possible etching problems of the ZnO film.^22^

Throughout this investigation, Al\ZnO\DLC-CF_*x*_ devices have demonstrated remarkable stability under accelerated aging conditions, including water jet erosion and UV exposure. Laser Doppler Vibrometry (LDV) analysis has verified the efficient SAW transmission through such a duplex coating system. To assess their performance, these protected systems were benchmarked against bare ZnO SAW devices, labeled as Al\ZnO and these devices coated with CYTOP, a commercially available fluorinated layer^38,39^ (Al\ZnO\CYTOP devices). Their deicing capabilities were tested for both supercooled sessile droplets and, under dynamic conditions, in an ice wind tunnel (IWT), revealing a superior performance for the bilayer protection approach. Thus, in addition to its remarkable protective role, we also demonstrated superior deicing efficiency for devices that implemented the bilayer. Specifically, a rapid deicing response. ensuring complete ice detachment in the IWT (∼30 s at −5 °C and a wind speed of 70 m/s), was achieved with minimal power consumption (5–7 W). Based on these findings, we present a critical analysis of power efficiency and propose a comprehensive model that accounts for the key physical factors influencing deicing processes in SAW devices integrated onto heat-conductive substrates.

## 2 Results and Discussion

### 2.1 Characteristics of DLC-CF_*x*_ Bilayers

DLC and CF_*x*_ coatings were prepared following previously optimized plasma enhanced chemical vapor deposition (PECVD) protocols for the individual thin films.^40,41^ Deposition was carried out sequentially within the same plasma reactor, at similar pressure and applied power conditions, to ensure a perfect integration between both layers and without exposing the DLC-CF_*x*_ interface to air. The detailed synthesis procedures are presented in the Experimental Section. The applications of room-temperature and solventless procedures, combined with careful tuning of the plasma conditions, ensure a full and conformal coverage of the substrates. The obtained DLC-CF_*x*_ coatings depict a homogeneous and compact morphology when deposited on a flat Si wafer or a fused silica substrate. The selected thicknesses of DLC bottom layer and CF_*x*_ top layer were ∼170 nm and ∼55 nm, respectively (Figure 1a), yielding a low root-mean-square roughness R_RMS_ value of 2.3 nm, as determined by AFM characterization of the layers deposited on Si reference substrates (Figure 1b).

**Figure 1:**
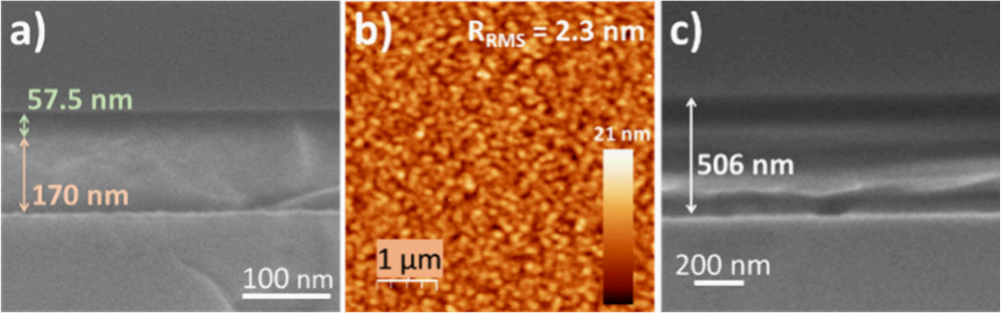
SEM and AFM micrographs of the protective coatings on reference substrates. (a and c) Cross-section SEM views of DLC-CF_*x*_ and CYTOP, respectively (arrows have been included to differentiate layer and substrate). (b) AFM micrography for DLC-CF_*x*_ deposited on Si.

The standard thickness of the CYTOP film used as a reference was set at approximately 500 nm (Figure 1c), i.e., the thickness of the fluorinated layer in the DLC-CF_*x*_ duplex coating is approximately one-tenth that of CYTOP. In this way, we aim to demonstrate efficient anti-icing capability while minimizing the amount of fluorine in the protective coating, in line with guidelines for reducing PFAS and fluorinated greenhouse gases already established by the European Chemicals Agency and EU regulations.^42^ Moreover, the synthesis of the fluorinated thin film through a vacuum process allows for the retention and controlled management of the waste generated. When the DLC-CF_*x*_ bilayer coating was deposited onto the Al\ZnO device, the surface roughness was substantially higher than on Si (Figure 2a). Thus, the fabricated SAW device surface presents R_RMS_ value of ∼26.1 nm (on the standard scale of 1 × 1 μm^2^), almost ten times that of the bilayer system on the reference substrate. Due to the low thickness of the DLC/CFx coating, this higher surface roughness must be linked to the roughness of the ZnO layer beneath.

**Figure 2:**
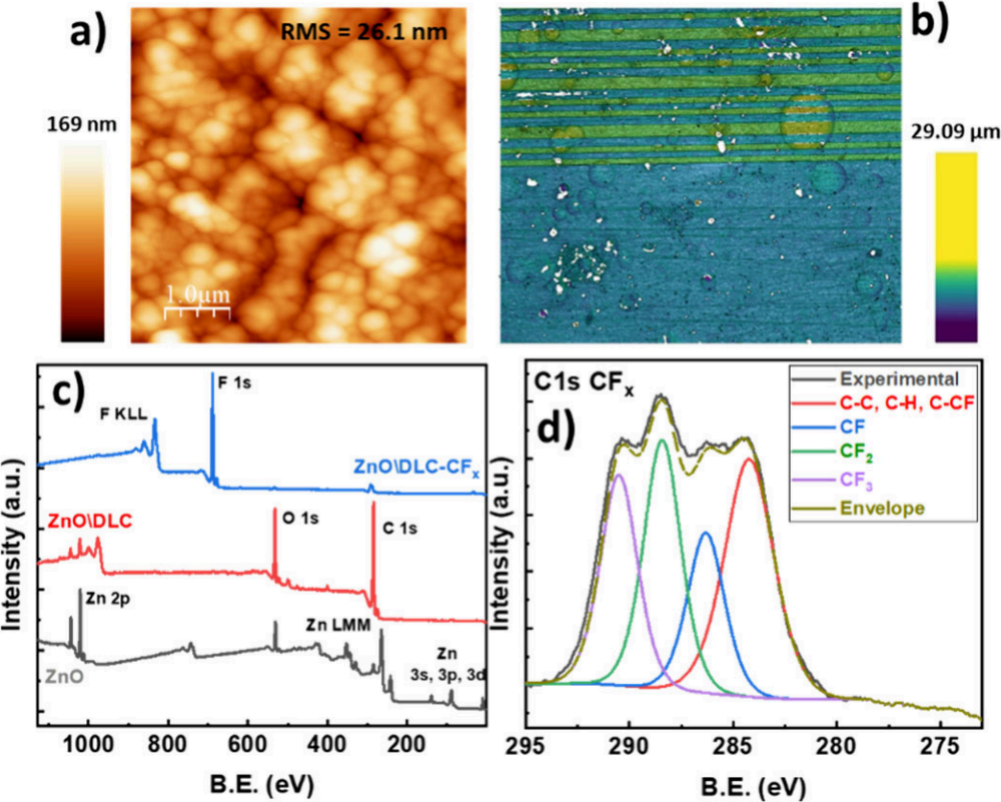
Surface morphology and chemical analysis of the DLC-CF_*x*_ devices. (a) AFM micrograph of the DLC-CF_*x*_ coated ZnO piezoelectric film on an aluminum substrate. (b) Confocal microscopy image of an Al\ZnO\DLC-CF_*x*_ chip acquired in the area close to the IDTs (emplaced at the top side of the image). (c) XPS general spectra recorded for ZnO, ZnO\DLC, and ZnO ZnO\DLC-CF_*x*_ bilayer, as labeled. (d) Fitting analysis of the C 1s photoelectron spectrum of the ZnO\DLC-CF_*x*_ surface.

As shown in Figure 2b, on a larger scale, the surface roughness of the Al\ZnO\DLC-CF_*x*_ is dominated by the surface features of the Al substrate, which is conformally covered by the ZnO film. This is so because the low thickness of the duplex polymeric-like coating and its capacity to efficiently cover any mound or asperity of the underneath film. Root mean square height (S_q_) and skewness (S_sk_) parameters of 0.53 μm 13.04 were deduced from the confocal analysis of the device surface near the IDTs (∼3 mm from the last finger).

The surface chemistry of the Al\ZnO\DLC-CF_*x*_ devices was assessed by X-ray Photoelectron Spectroscopy (XPS). Figure 2c shows the survey spectra obtained for Al\ZnO, Al\ZnO\DLC, and Al\ZnO\DLC-CF_*x*_ devices. They exhibit peaks corresponding to Zn, O, and C in the sample Al\ZnO, and a carbon-rich surface characteristic of diamond-like carbon (DLC) for the Al\ZnO\DLC. In this case, little peaks corresponding to Zn were still observed in the XPS survey spectrum. As the DLC is a very compact film on top of most substrates, the presence of the ZnO 2p photoemission peaks implies that due to its protuberances and relatively high roughness there are zones where the DLC is not perfectly covering all the ZnO film surface. The spectrum of the bilayer only depicts the characteristic features for CF_*x*_,^33,35^ indicating predominant contributions from C and F peaks, with no traces of Zn detected. This confirms that the upper surface of the device is entirely covered by the bilayer, with no signs of ZnO. This is indicative of the suitability of the selected PECVD protocol, which clearly avoids etching issues in the piezoelectric layer and leverages the conformal formation of a compact DLC-CF_*x*_ duplex coating.

A detailed analysis of the chemical states of the DLC and CF_*x*_ layer surfaces was further performed by comparing the C 1s spectra for the ZnO\DLC-CF_*x*_ (see Figure 2d) and ZnO\DLC samples (Figure S1 and Table S1 in the Supporting Information). In good agreement with data reported in refs 33,43 , the C 1s peak is deconvoluted into C–C and C–H bonds for the DLC. Meanwhile, the fitted peak components for the C 1s spectrum of the ZnO\DLC-CF_*x*_ (in Figure 2d) are attributed to the following functional groups: −CF_3_ (291.0 eV, 8.6%), −CF_2_– (288.9 eV, 10.4%), −CF= (286.7 eV, 8.3%), and C–H, C–C, C–CF (284.8, 9.7%).^44^ The chemical composition quantification reveals that the surface is composed of 67.40% fluorine, 30.80% carbon, and 1.80% oxygen, expressed in terms of atomic concentrations. The abundant presence of fluorine with respect to oxygen reveals that C–F_*x*_ bonds are dominant on the surface and confirms the fluorinated character of the CF_*x*_ upper layer and its capacity to completely cover the surface. It is noteworthy that a similar surface composition and distribution of CF groups is typical of the CYTOP coatings prepared by wet routes, with the difference that the CYTOP’s C 1s spectrum exhibits a relatively larger contribution of −CF_2_ groups.^44^ This indicates that fluorocarbon chains in the CYTOP layer are probably longer and less branched than in the PECVD CF_*x*_ thin film.

### 2.2 Wettability and Freezing Delay Time of Pristine Devices

Table 1 lists the dynamic contact angles and sliding angles measured at 22 °C for the surfaces of the pristine Al\ZnO, Al\ZnO\CYTOP, and Al\ZnO\DLC-CF_*x*_ devices. Static wetting angles and freezing delay times determined at −5 °C are also listed in this table. It is noteworthy that the Water Contact Angles (WCA) for the latter two chips were reproducible over time; however, the wetting properties of the Al\ZnO chips were continuously changing. Thus, immediately after preparation, the ZnO surface was highly hydrophilic, but then its WCA increased to reach the values listed in Table 1 after one month of storage in the dark. We and others have experienced this wettability variation for different metal oxides, including ZnO and TiO_2_, prepared by PECVD and magnetron sputtering.^45,46^ We attribute this phenomenon to various factors, including the generation of radicals and the activation of the surface by the oxygen plasma and UV exposure, which are inherent to the plasma-assisted deposition of oxide films, as well as to posterior differences in their degree of surface hydroxylation and airborne carbon contamination of the as-grown surfaces exposed to air.

**Table 1:** Dynamic Contact Angles (*θ*_adv and_ *θ*_rec_), Sliding Angles (Δ*θ*) measured at 22 °C, Static Wetting Contact Angles (*θ*) and Freezing Delay Times (FDTs) at −5°C measured on “As-Prepared” devices and after Aging Tests for ZnO, ZnO\DLC-CF_*x*_ and ZnO\CYTOP chips.

As-prepared devices						Aged devices	
Device surface	θ_adv_	θ_rec_	Δθ	θ (−5 °C)	FDT(−5 °C) (min)	θ (water jet)	θ (UV)
ZnO[Table-fn t1fn1]	57 ± 3	50 ± 3	7 ± 3	38 ± 4	10	<5	<5
ZnO\DLC-CF_*x*_	100.4 ± 0.5	92 ± 4	8.5 ± 0.5	75 ± 2	89	104 ± 1	17 ± 1
ZnO\CYTOP	110 ± 1	104.3 ± 0.5	5.4 ± 0.5	71 ± 2	30	20 ± 1	<5

1 The values included in the table correspond to pristine samples stored at room conditions for one month (see text).

Wetting parameters in Table 1 indicate that at ambient temperature, the surfaces of the CYTOP and DLC-CF_*x*_ devices are hydrophobic, exhibiting low hysteresis and small sliding angles. These characteristics facilitate the easy roll-off of water droplets, indicating a low adhesion of water on the fluorinated surfaces. For the three coated systems, the WCA values were lower at subzero temperatures, a common trend observed in water droplets on a wide variety of materials due to a Cassie–Baxter to Wenzel transition driven by water vapor condensation.^32,47,48^

Freezing Delay Time (FDT) values in Table 1 follow the increasing order of Al\ZnO, Al\ZnO\CYTOP, and Al\ZnO\DLC-CF_*x*_. The highest value found for the Al\ZnO\DLC-CF_*x*_ device can be taken as an indication of an effective anti-icing behavior.^32,48–50^ Conversely, the lower FDT value found for the Al\ZnO\CYTOP device suggests a less pronounced anti-icing capacity. Tentatively, we associate this feature with a certain surface inhomogeneity and the predominance of relatively longer −CF_2_– chains in the CYTOP sample than in the CF_*x*_ plasma thin film, a feature that could favor the nucleation of ice on the CYTOP surface.

### 2.3 Environmental Durability: Water Jet and UV Irradiation

The effects of the accelerated aging tests on the wetting behaviors of three types of chips are summarized in Table 1. Meanwhile, Figure 3 showcases their surface morphology states and the effect on WCA of the UV irradiation.

**Figure 3:**
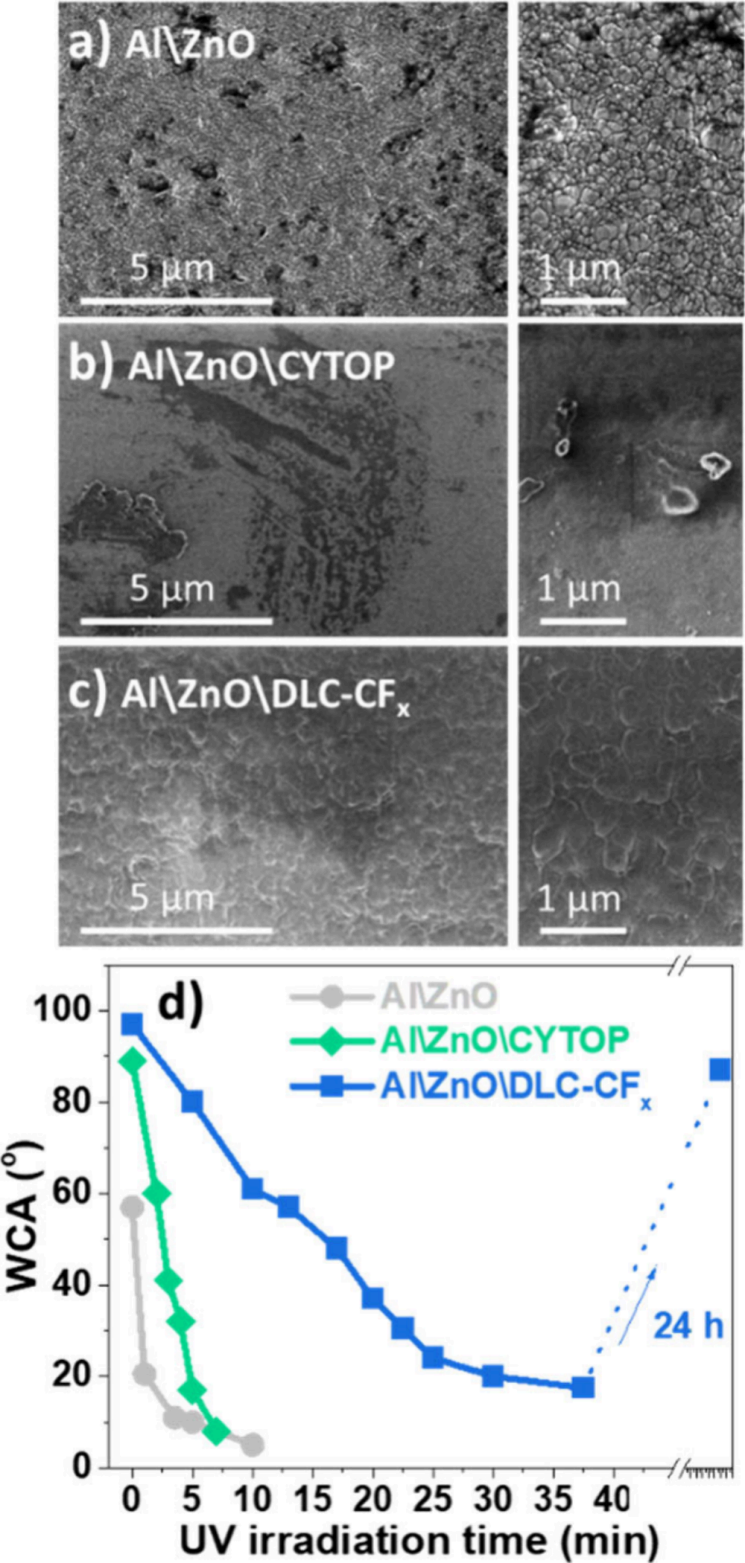
Analysis of environmental stability of ZnO, ZnO\CYTOP, and ZnO\DLC-CF_*x*_ surfaces. Normal-view SEM micrographs of the surface of the Al\ZnO (a), Al\ZnO\CYTOP (b), and Al\ZnO\DLC-CF_*x*_ (c) devices after exposure to a water jet for 3 h. The stains in a) and b) correspond to damaged zones and local delamination at the surface. (d) Evolution of the WCA of the Al\ZnO, Al\ZnO\CYTOP, and Al\ZnO\DLC-CF_*x*_ surfaces exposed to high-intensity UV irradiation as a function of time and, for the latter sample, recovery after its handling in air for 24 h.

The aging experiments for the Al\ZnO chips were always carried out when the as-prepared device had a water contact angle of approximately 50° (i.e., moderately hydrophilic state). This wetting angle drastically changed when the chips were subjected to the water jet test for 3 h, transforming the surface state into superhydrophilic (WCA < 5°).

It should be noted that changes in the WCA were already observed for shorter water jet exposures. These Al\ZnO chips slowly evolved over time to recover the initial WCA value of 50°–60° after storage for one month in dark conditions. Besides changes in WCA, water jets induced slight but detectable changes in the morphology and appearance of ZnO surfaces as determined by SEM. Figure 3a shows a micrograph of the surface morphology of the Al\ZnO chips after the water jet test. Apart from the characteristic compact arrangement of nanocolumns for polycrystalline ZnO, a significant number of dark stains, as well as a more blurred aspect than for the original ZnO surfaces can be observed comparing the surface micrographs of aged and pristine ZnO surfaces (see SEM micrographs of pristine ZnO in Figure S2). These modifications of the SEM images suggest that the ZnO surface has experienced morphological and, likely, compositional changes. After one month of storage following the water jet test, the surfaces of the Al\ZnO chip were exposed to UV irradiation using a high-intensity lamp. Figure 3d shows that exposure for a few minutes resulted in the transformation of its surface state to superhydrophilic with a contact angle value lower than 10°.

When the Al\ZnO\CYTOP chip was subjected to 3 h of water jet exposure, it exhibited a drastic WCA decrease from 109° to 20°, although after one month of storage in the dark, the WCA was recovered to 90°. When this same chip was exposed to a high-intensity UV lamp, it became hydrophilic in less than 10 min (see Figure 3d). The WCA was recovered over time after UV illumination, following a trend similar to that observed for ZnO. We attribute all these features to the partial degradation of the Al\ZnO\CYTOP external surface,^38,44^ as suggested by the SEM analysis of the surface of the aged chips (c.f., Figure 3b), showing the appearance of defect features in the form of stains, some rippled structures, and local delamination, the latter likely due to a poor adhesion to ZnO.^50^ Such degradation was confirmed by the XPS analysis of the surface state of the sample, showing a high percentage of functional groups with C–O and O-H bonds, , a very high concentration of C–H and C–C groups, and a distribution of carbon–fluorine bonds with a high predominance of CF_2_ groups (see Supporting Information Figure S3 and Table S2).

Interestingly, Al\ZnO\DLC-CF_*x*_ chips subjected to the water jet test for 3 h depicted similar WCA values of ∼ 104° before and after the test. Moreover, the topography of the surface state of the Al\ZnO\DLC-CF_*x*_ chip, as analyzed by SEM, did not show significant changes after the tests (c.f. Figure 3c). The robustness of the surface morphology of these chips after the water jet and UV irradiation tests was also confirmed by assessing their roughness relative to that of the other two chips. The S_sk_ value,^51^ a long-range roughness parameter which is typically used to assess the contribution of hills or valleys above or below a reference surface plane, is a useful tool to depict the evolution of device surfaces subjected to erosion tests. As deduced from the analysis of confocal microscopy images (see Figure S4 and Table S3), the pristine Al\ZnO chip showed a S_sk_ value greater than zero, indicating that peaks predominate over valleys. In contrast, the aged Al\ZnO reveals a certain flattening on its surface with a S_sk_ value slightly below zero. Since the roughness of these devices is primarily due to the aluminum substrate, the three devices exhibited similar roughness in their pristine state. Therefore, a greater value of S_sk_ in devices coated with a protective layer, compared to the Al\ZnO device, evidences that in a certain manner the protective layer does not alter the original roughness of the device. Both the Al\ZnO\CYTOP and Al\ZnO\DLC-CF_*x*_ devices show S_sk_ values above zero, the latter possesing the highest value and thus having a roughness quite similar to that of the device without coating.

The WCA for the Al\ZnO\DLC-CF_*x*_ sample also decreased after the UV irradiation treatment (c.f., Figure 3d), but at a much slower pace than the Al\ZnO and Al\ZnO\CYTOP devices. The Al\ZnO\DLC-CF_*x*_ sample required an irradiation for more than 35 min to reach an almost stable hydrophilic state (ca. 17°). Then, a relatively fast recovery occurred in the dark, with its WCA reaching a value of ca. 85° just 24 h after the irradiation test. This behavior suggests that the decrease in the WCA upon high-intensity UV irradiation is due to surface hydroxylation induced by the breaking of some C–F and C–C bonds at the outermost surface of the coating. This process can promote some reversible surface adsorption of H_2_O and/or OH- groups^52,53^ but leave the DLC-CF_*x*_ duplex coating unaltered. This was supported by the XPS analysis of this chip, which showed that after the aging tests, the C 1s spectrum closely resembled that in Figure 2d, recorded for the pristine sample.

All these evidence support that the Al\ZnO\DLC-CF_*x*_ chips are functionally adaptable to simulated environmental conditions and more resilient than the bare Al\ZnO chips and the commercial CYTOP coated Al\ZnO\CYTOP chips. Good adhesion and stability, together with the capacity of the thin DLC-CF_*x*_ duplex coating to completely cover the surface, are key factors in the protective performance of these coatings.

### 2.4 Acoustic Wave-Field of Coated and Uncoated Devices

SAW devices based on Al\ZnO were analyzed electrically using a Vector Network Analyzer (VNA), and their acoustic wave field was examined using a Laser-Doppler Vibrometer (LDV) (Figure 4). From the reflection spectra obtained from the VNA, operation frequencies for Al\ZnO, Al\ZnO\DLC-CF_*x*_ and Al\ZnO\CYTOP devices were determined to be 23.24 MHz, 27.35 MHz, and 23.22 MHz, respectively. The variation in frequency among devices is mainly due to the differences in the IDT layouts, as gathered in . The |S_11_|^2^ curves measured for these two devices are presented in Figure 4a, while the averaged amplitudes of the excited SAWs are shown in Figures 4b and c. These representations of the amplitudes confirm that all IDTs enable the excitation of similar SAWs and consequently should yield equivalent deicing effects.

**Figure 4:**
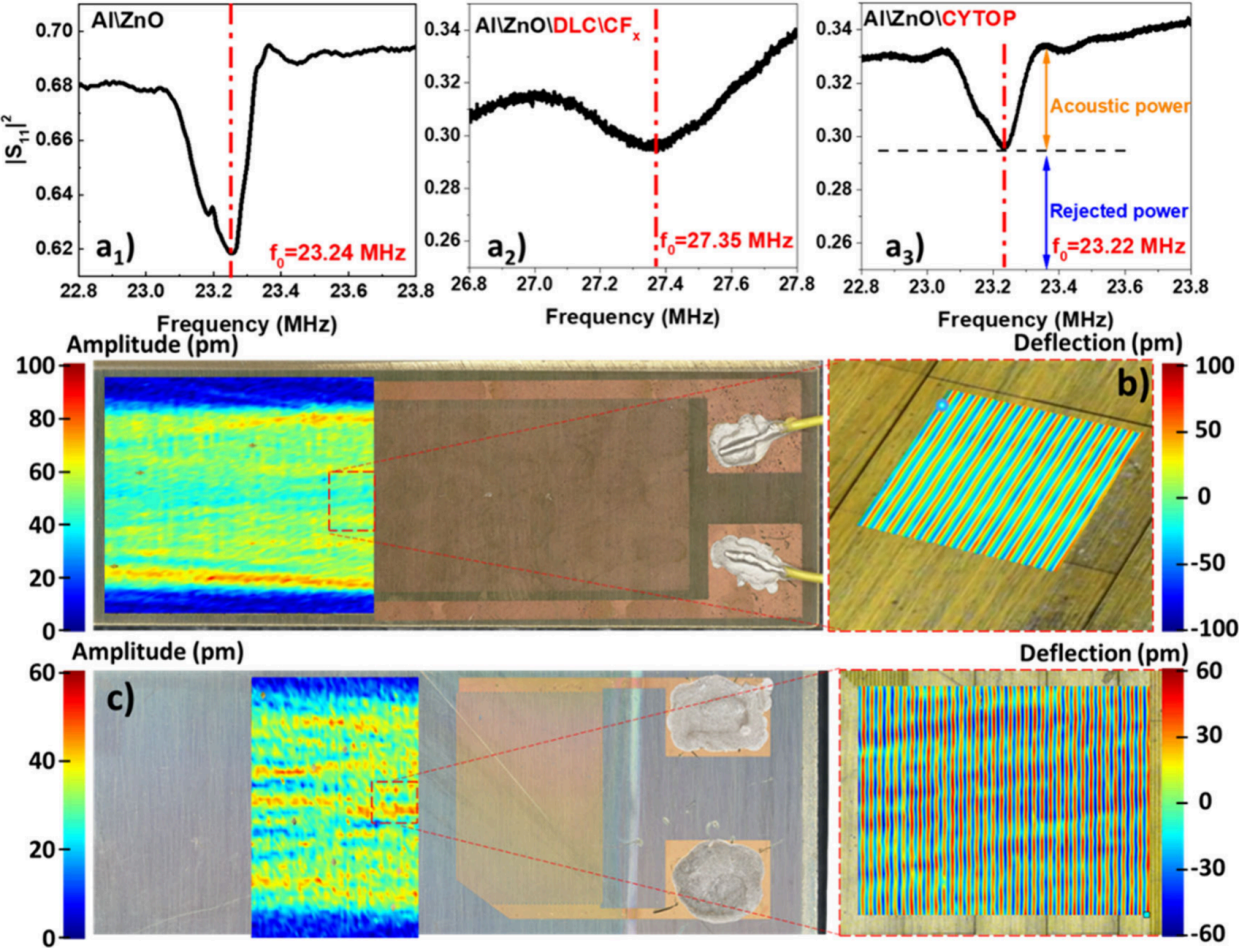
Comparison of the radio frequency and acoustic behavior of the chips: Al\ZnO (a_1_, b), Al\ZnO\DLC-CF_*x*_ (a_2_, c) and Al\ZnO\CYTOP (a_3_) devices. (a) Power reflection coefficient |S_11_|^2^ determined using the vectorial network analysis. (b–c) Surface-normal amplitude distribution in the form of color maps (overview) and snapshot of the surface deflection in front of IDT (inset) determined by DLV Laser-Doppler Vibrometry for (b) Al\ZnO and (c) Al\ZnO\DLC-CF_*x*_.

Figure 4a shows a more intense peak for the Al\ZnO device than for the chips with protective layers, which can be attributed to the different IDT layouts and/or the effect of the coatings themselves. In particular, we attribute the more rounded and wider peak shape found for the Al\ZnO\DLC-CF_*x*_ chips to its IDT design consisting of fewer fingers than in the other two cases (see Experimental Section). The pure acoustic power, Joule effect, and capacitive losses contributions have been taken into account to estimate the effective power of SAWs. As indicated in the panel for the Al\ZnO\CYTOP chip in Figure 4a, the effective acoustic power has been taken as the difference between the effective power outside the acoustic peak and inside the acoustic peak. The |S_11_|^2^ values at the minimum of the curves have been used to calculate the effective power using eq 1, explained in the Experimental Section. Thus, power loss, reflected power, and power converted to SAWs could be derived. We found that around 31%, 55% and 60% of the input power leads to ohmic or capacitive losses for the devices of Al\ZnO, Al\ZnO\DLC-CF_*x*_, and Al\ZnO\CYTOP, respectively. These values are important for the deicing efficiency, which appears to be based on both Joule heating and acoustic interaction. Similarly, large portions of the input power are reflected back to the amplifier, i.e., about 62% for the Al\ZnO sample and about 30% in both Al\ZnO\DLC-CF_*x*_ and Al\ZnO\CYTOP samples. In turn, the portion of the input power converted to SAWs amounts to about 8% in the Al\ZnO sample and about 4% for the samples with a coating. These values appear relatively low when compared with the values obtained by IDTs activation ofsingle-crystalline samples such as LiNbO_3_ plates,^12^ but are within the typical values found for long-wavelength IDTs on ZnO substrates. These relatively lower values are attributed to the dispersive nature of piezoelectric thin film polycrystalline devices and their low coupling coefficient. In addition, the smaller number of fingers in the IDT of the Al\ZnO\DLC-CF_*x*_ chip is likely contributing to acoustic-wave losses in this case. Despite these limitations, the obtained results show that the combination of the high environmental resilience depicted by the Al\ZnO\DLC-CF_*x*_ chips and the preservation of sufficient SAW transmission open the way for the implementation of ZnO-based piezoelectric devices for outdoor applications.

### 2.5 Ice Detachment and Deicing by Surface Acoustic Wave (SAW) Activation

Ice adhesion tests upon SAW activation of the uncoated and coated chips were carried out at the freezing chamber, i.e., under static conditions, following the experimental procedure detailed in the Experimental Section and Figure S6. Figure 5 shows the dispersion of times required to detach the ice probe subjected to a tension of 4 N as a function of the power applied to the Al\ZnO, Al\ZnO\CYTOP and Al\ZnO\DLC-CF_*x*_ devices (note that this force is equivalent to an applied tensile stress of 52.38 kPa). The results indicate that the detachment of ice from the Al\ZnO\DLC-CF_*x*_ chips requires significantly less SAW power and time. Thus, the ( ice detachment from this device occurred at effective power levels lower than 0.5 W). This finding is consistent with the expected fast ice removal from low surface energy materials, particularly those that are highly hydrophobic, such as CF_*x*_ and Teflon-like polymers.

**Figure 5:**
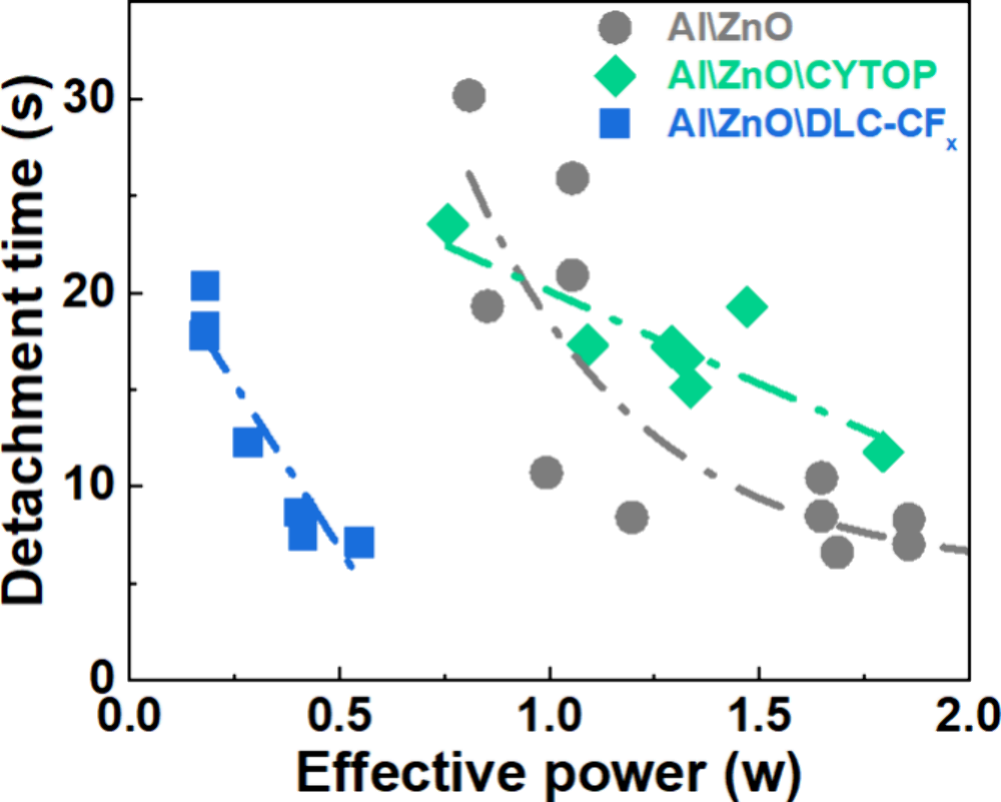
Ice detachment and AW effective power. Ice probe detachment times as a function of AW effective power for the Al\ZnO, Al\ZnO\CYTOP, and Al\ZnO\DLC-CF_*x*_ SAW devices. Lines are drawn to guide the sight.

In contrast, both the effective powers and required times were substantially larger/longer for the Al\ZnO and Al\ZnO\CYTOP devices compared to those for the Al\ZnO\DLC-CF_*x*_ device. A higher power for the Al\ZnO chip is consistent with the fact that ice forms directly on the rough and hydrophilic surface of ZnO. On the other hand, SEM micrographs of the Al\ZnO\CYTOP chip surface after the first detachment experiment revealed a circular mark along the perimeter of the ice probe (as shown in the low-magnification micrographs in Figure S7), also showing other marks and stains suggesting some degradation of the CYTOP coating.

This suggests that the CYTOP coating was either removed or damaged in the area covered by the probe, and that the CYTOP-ZnO interface is not robust upon ice detachment under the test conditions. This agrees well with the observations from the aging tests in Section 2.2. The trends shown in Figure 5 for the detachment times of the three systems can be interpreted assuming that the detachment of the ice probe requires the melting of a thin layer of ice at the interface. This detachment process seems to be more effective for the Al\ZnO\DLC-CF_*x*_ chip. It is thus likely that the stable and hydrophobic surface of the DLC-CF_*x*_ duplex coating effectively reduces the ice adhesion strength because wetting of the CF_*x*_ surface termiantion by the water interface layer is not a favored process. We also hypothesize that the cracking and partial melting involved in the softening of the ice-substrate interface^34^ are more effective for the DLC-CF_*x*_ than the CYTOP termination. In the former case, the high elastic constant and low mass density of the DLC would enable a more efficient AW transmission.^54^ In addition, a lower attenuation of SAW transmission through the coating is to be expected due to the much lower total thickness of the bilayer in comparison with that of the CYTOP coating.

### 2.6 Deicing of Glaze Ice by SAW in Static Conditions

Experiments were also conducted to investigate the SAW-induced melting of small glaze ice aggregates formed at −15 °C under static conditions, i.e., in the freezing chamber upon freezing of water sessile drops (see Experimental Section). Figure 6 shows a series of snapshots taken from a video of the melting process (see also Videos S1 and S2) of a glaze ice aggregate placed on a pristine Al\ZnO\DLC-CF_*x*_ chip when a power of 5.0 W was applied (i.e., with an effective power of 3.56 W due to a power loss of 30%). From t = 1.00 s to t = 4.39 s, the SAW activation did not alter the aspect of the ice aggregate but produced the removal of the frost accumulated on the surface of the chip during its cooling in the chamber. From t = 4.39 s to t = 5.37 s, the ice aggregate began to melt, as evidenced by a slight change in the WCA to values slightly smaller than 90°. Despite this initial change at the bottom of the aggregate, its characteristic tip shape remained unchanged. This indicates that the melting process has not yet reached the top part of the aggregate and is localized at the interface. Only at t = 17.57 s did this characteristic tip disappear. Finally, for longer times up to t = 28.75 s, some small particles of ice still remaining in the interior of the melted water droplet completed their melting. This suggests that before the dissipated heat has time to effectively diffuse throughout the entire droplet, the streaming effect of the acoustic wave causes the droplet-air interface to melt and therefore provokes the disappearance of the tip before that complete melting of the ice droplet takes place. A similar behavior was observed for a pristine Al\ZnO\CYTOP device, although the total time required for melting was shorter in this case (see the Supporting Information, Figure S8).

**Figure 6:**
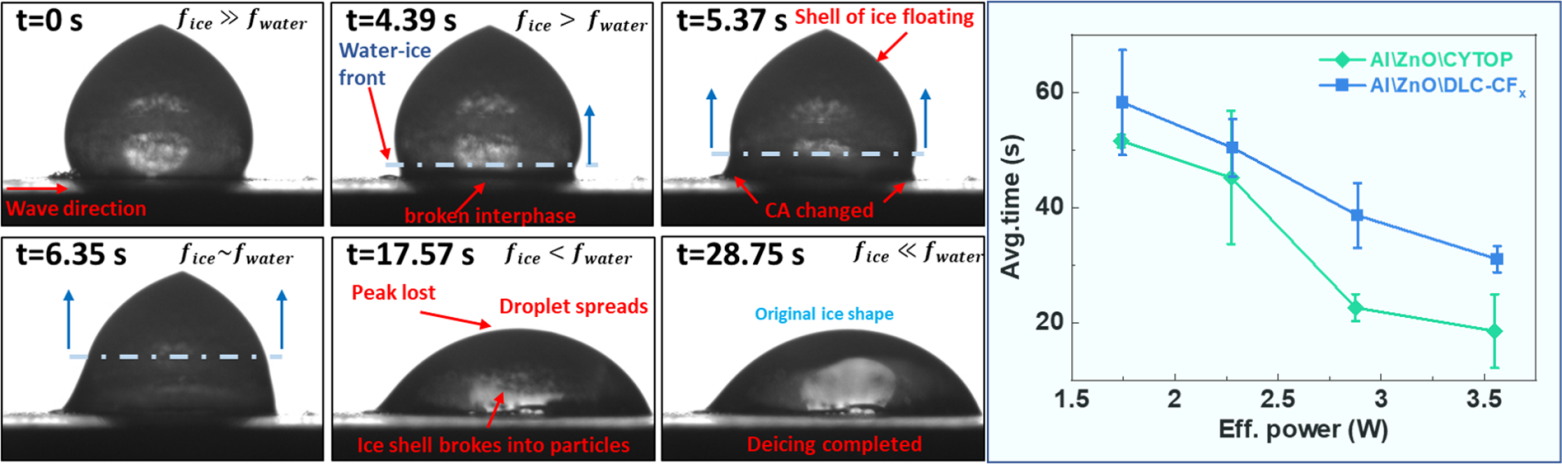
SAW-induced melting of small glaze ice aggregates under static conditions. (Left) Snapshots of a small glaze ice aggregate formed under static conditions during activation of an Al\ZnO\DLC-CF_*x*_ chip at an effective applied power of 3.56 W. (Right) Melting time of glaze ice aggregate as a function of effective power comparing Al\ZnO\CYTOP and Al\ZnO\DLC-CF_*x*_ devices. Reported data are the average of at least three experiments for each effective power; the error bars define the spam of variation found in each case.

In previous studies on SAW deicing, it was found that the interface melting progression is a relevant deicing mechanistic step for small glaze ice aggregates placed directly atop the IDTs.^7,10^ This process was attributed to several physical effects, including the cracking of ice at the interface by the mechanical impulses of the SAW, followed by the melting of ice induced by the interface heating due to the thermal energy provided by acousto-thermal effects and/or by ohmic losses in the form of Joule resistive effects and/or other capacitive losses at the IDT. For the devices prepared on efficient heat-conductive aluminum substrates, as those investigated in the present work, thermal effects are expected to play a significant role in the SAW-induced deicing. The panel on the right side of Figure 6 shows that melting time, besides decreasing with the effective power, also depends on the type of exposed surface on each chip, either CYTOP or DLC-CF_*x*_. Thus, on the Al\ZnO\CYTOP chip, deicing was faster for all applied powers, an effect that we link with the higher hydrophobicity of the surfaces of this type of devices both in their pristine state (c.f. Table 1) and after deicing activation (i.e., ∼90° vs ∼60° for CYTOP and DLC-CF_*x*_, respectively).

Since the comparison between Figures 6 and S8 reveals that the deicing mechanism proceeded through equivalent stages on the Al\ZnO\CYTOP or the Al\ZnO\DLC-CF_*x*_ chips, we link the different deicing times with the magnitude of WCAs on the two surfaces.

### 2.7 Deicing in IWT Dynamic Conditions

Deicing experiments of large aggregates of ice were carried out on pristine Al\ZnO\DLC-CF_*x*_ and Al\ZnO-CYTOP devices activated by SAWs in an IWT mimicking more realistic conditions for ice accretion (see Videos S3 and S4 and details on the experimental setup in Figure S9). Figure 7 shows selected snapshots for a 45° orientation of the devices with respect to the wind flow. Videos were recorded with visible and infrared (IR) cameras at different stages of the deicing process of glaze ice accreted at −5 °C. Selected snapshots correspond to t = 0 s (Figure 7a1 and b1) and at several intermediate stages (Figure 7a2/a3/a4 and b2/b3/b4) of the deicing process up to the complete detachment of the ice aggregates (Figure 7a5 and b5), occurring after 32 s and 35.6 s for the Al\ZnO\DLC-CF_*x*_ and Al\ZnO\CYTOP devices, respectively.

**Figure 7:**
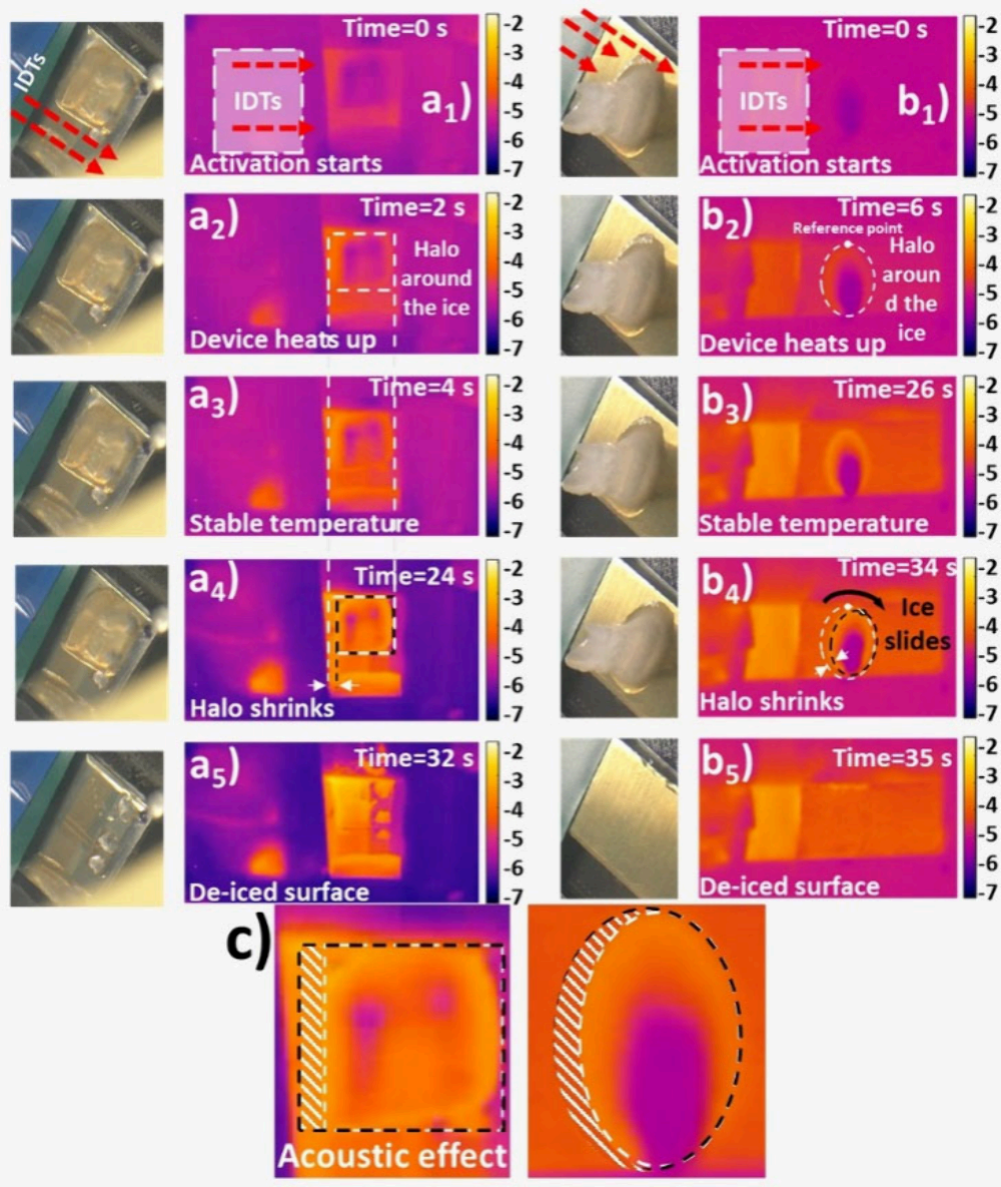
SAW induced deicing of large ice aggregates in the IWT for the Al\ZnO\DLC-CF_*x*_ (a_i_) and Al\ZnO\CYTOP (b_i_) at −5 °C for the devices placed at an angle of 45° with respect to the wind flow (wind velocity of 70 m/s). a_1_–a_5_/b_1_–b_5_ snapshots taken at the indicated times from the visible and infrared videos recorded during the deicing process. Specific features of the ice aggregates and some small modifications induced in them during the deicing are indicated in the snapshots. (c) Enlargement of snapshots a_4_ and b_4_ to evidence the loss of perimeter area of the ice aggregates as a result of the activation process: black and white perimeter dotted lines represent the profiles of the ice aggregate in a_2_/b_2_ and a_4_/b_4_, respectively.

The IR images in Figure 7 (right side of the panels) provide additional information about the deicing mechanism. The evolution in brightness and color in the IR snapshots in Figure 7a_2_/a_3_ and b_2_/b_3_ indicates that the temperature measured at the bottom edge perimeters of the ice aggregates was progressively increasing with time. This effect was even more evident for experiments carried out with rime ice at −15 °C on the Al\ZnO\CYTOP chip (see Supporting Information, Figure S10). In subsequent intermediate snapshots in Figure 7, it is also apparent that the profiles of ice aggregates undergo a certain reduction in size (Figure 7a_4_ and b_4_, and Figure 7c for an enlarged view of these snapshots) before finally displacing upward and becoming detached from the surface (Figure 7a_5_ and b_5_). Sliding and detachment events were generally separated by 2–3 s, but in many cases the sliding stage was not observed, and detachments took place in a sudden way (see Videos S5 and S6). This occurred even in experiments where the chips were oriented at 100° with respect to the wind direction. We attribute the sliding and detachment process to the reduction of the ice-device adhesion force due to the partial interface melting, followed by the aggregate removal under the force action of the wind impinging on the chip surface. These IWT experiments also support that the hydrophobic character of the surfaces of DLC-CF_*x*_ and CYTOP, in their pristine state, facilitates the detachment of ice aggregates.

The enlarged IR snapshots in Figure 7c (corresponding to panels a_4_ and b_4_) provide additional information about the interface activation process. The reduction of the lateral area of the edge profile of the IR images in Figure 7c) is a general behavior found in all experiments, suggesting that SAW activation and ice interface melting occurred first, and were particularly pronounced, at the side facing the IDTs. In previous works, we demonstrated that the low-wavelength oscillations of Rayleigh SAW are effectively attenuated through the activation of the ice-substrate interface within a distance of approximately 10 wavelengths.^12,13^ Herein, 10 wavelengths are equivalent to more than 1 mm, precisely the reduction in the size of ice aggregates estimated from the analysis of the brightness and color distribution of IR images in Figure 7c). Figure 8 shows the estimated average energy E (calculated as E = P_eff_t_eff_) required for deicing of the two devices at various testing temperatures (−5 and −15 °C) and at the two angles of wind attack (AoA).

**Figure 8:**
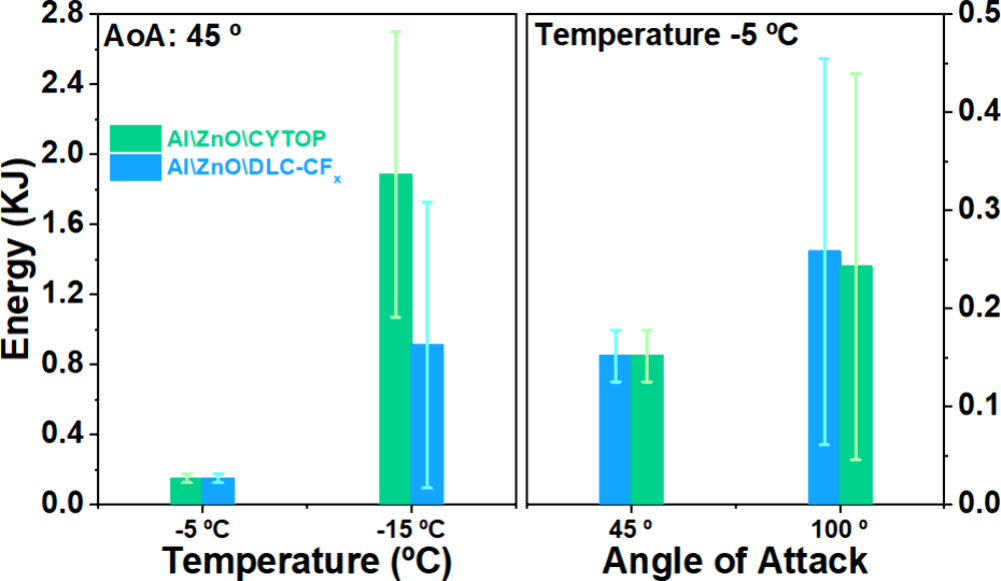
Average energy consumption in IWT deicing experiments depending on the test temperature. energy consumption of DLC-CF_*x*_ and CYTOP acoustic devices on tests for the two different temperatures and angle of attach (AoA) of 45° (left), and for two AoAs at a fixed temperature of −5 °C (right), working with a wind speed of 70 m/s. Column values are the average of four experiments for each test. The error bar accounts for the large spam of values determined in each case.

Although the large dispersion of results in this series of experiments precludes a quantitative comparison of test results, some general, semiquantitative trends can be deduced from the data in Figure 8. First, a significant increase in energy was found for the experiments at −15 °C with respect to those at −5 °C (both for an orientation of 45°). This significant difference highlights the importance of thermal effects in the ice removal processes and suggests that the heat released into the air may slow down the interface softening process. Second, a high energy was required for deicing with the Al\ZnO\CYTOP chips at −15 °C, an increase that we associate with the progressive degradation of the surface state of this sample along successive experiments and the resulting growth of the ice adhesion force. Third, the wider error bars in energy required for deicing at an angle of 100° underline the importance of wind dynamics in the deicing process. In particular, the effect on the reproducibility of the experiments of the strong turbulent components in the air flow close to the surface of the sample. Clearly, the affectation of experimental results by the selected variables points to the fact that both the cooling of the chips by the air flow and the wind dragging force acting on the aggregated ice are crucial in controlling ice detachment.

### 2.8 Duplex Coating of SAW Devices and Mechanism of Deicing

On dielectric or low heat-conducting piezoelectric substrates, the mechanism of deicing induced with Rayleigh-SAWs proceeds through the melting of a small ice zone at the edge facing the IDTs, followed by the lateral progression of a waterfront up to complete melting of the ice.^12,13^ The results in Figures 7 and S10 suggest that, following the SAW activation of the lateral zones of the ice aggregates facing the IDT, ice removal by detachment occurs before complete melting has taken place. Results in Figures 5 and 6 further support this mechanistic view, proving that, in addition to the initial SAW activation, interface melting along the entire contact area effectively contributes to the observed droplet melting and ice detachment, respectively. We hypothesize that this thermal contribution may occur over relatively large areas because the high thermal conductivity of the Al substrate helps to homogenize the heat distribution throughout the entire system. The thermal conductivities of the materials in contact at the water/ice/ZnO/Al interfaces follows the order of water < ice ≪ ZnO ≪ Al, with averaged values of 0.5 W/(m·K) for water,^55,56^ 2.2 W/(m·K) for ice,^55,56^ 60 W/(m·K) for ZnO,^57^ and 220 W/(m·K) for aluminum.^58^ Here, we neglect the possible barrier effect of the DLC-CF_*x*_ coating because of its very small thickness and the high variability of the thermal conductivity of DLC with composition and temperature.^59^ These values support the hypothesis that during SAW activation, the aluminum substrates will act as an effective heat sink and distribute any thermal load produced in the system, either by acoustothermal effects,^10,60,61^ or by Joule effects due to imperfections in the IDTs.

Taking the experimental evidence and these thermal conductivity values into account, Scheme 2 summarizes the factors involved in the SAW-induced deicing mechanism when the piezoelectric films are deposited on a highly heat-conductive substrate. This situation differs significantly with respect to previous studies carried out by our group on glass or bulk piezoelectric substrates, where heat conductivity is much smaller than in the present case.^12–14^ Scheme 2a indicates that, as previously stated in those works, the SAW reaching the ice edge becomes attenuated within a short interface region from the ice edge. This attenuation length has been estimated in about 10 wavelengths for LiNbO_3_ substrates,^12,13^ a distance that in the current experiments would amount to more than 1 mm. In this region, ice cracking and partial melting due to the mechanical activation of the interface are prevalent, particularly at the beginning of the deicing process. This situation corresponds to the experimental images a_1_–a_4_ and b_1_–b_4_ in Figures 7a and 7b. Scheme 2b shows that heat will be simultaneously dissipated and distributed rather rapidly along the full aluminum substrate, contributing to the heating of the entire ice-device interface and promoting partial ice melting over a long interface area, as well as the eventual detachment of the entire ice aggregate under IWT conditions (cf., Figure 7a_5_ and b_5_). Thus, besides its higher stability and resilience under harsh conditions, the DLC-CF_*x*_ coating plays an important role in favoring the deicing process through the hybrid procedure outlined in Scheme 2. First, DLC is compatible with the transmission of SAWs, while improving wear and environmental resistance. Second, the thin duplex coating imposes no meaningful restriction on heat transmission from the substrate. Third, its high stability and the ice-phobic character of the CF_*x*_ ensure that the adhesion force of the layer of water formed at the interface is relatively weak on this water-repellent surface. The WCA/FDT values listed in Table 1 and the preferential detachment found during the ice detachment tests in Figure 5, clearly support this view.

**Scheme 2:**
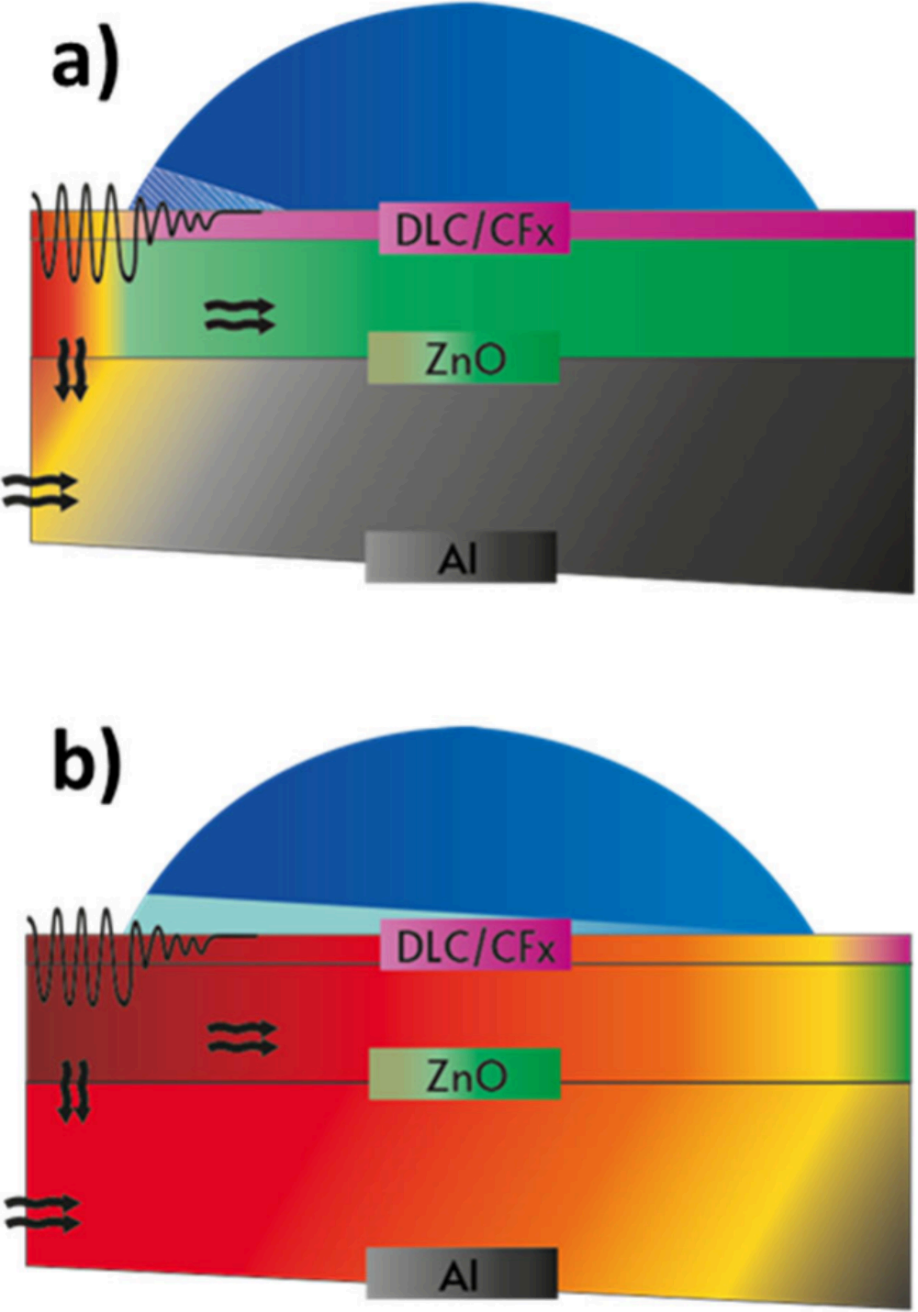
Schematic Description of the Short Wavelength SAW Induced De-Icing Mechanism Using ZnO Based Devices Deposited on High Thermal Conductive Substrates: (a) Initial Stages of the Activation Process;^a^ (b) Final Stage Just before the Detachment of Ice^b^

a The SAW becomes attenuated at a small distance within the ice/coating interface, inducing localized cracking and, possibly, partial melting in this zone. Simultaneously, the heat produced by the acoustothermal effect propagates from the ZnO into the aluminum; additional heat coming from the IDTs will sum up this effect in real experiments.
b The heat produced at the ice/water edge at the side facing the IDTs and the other sources of heat existing in the system are effectively distributed through the aluminum plate, producing the melting at the ice/coating interface. At this stage, ice adhesion to the coating is drastically reduced, and detachment occurs. The arrows indicate the flux of heat occurring during the de-icing process. Red and yellow colors indicate the device parts progressively heated up during the experiment

## 3 Conclusions

We have demonstrated that the surfaces of ZnO employed for monolithic surface acoustic wave deicing are significantly affected in terms of surface integrity and, particularly, wetting behavior when exposed to harsh environmental conditions, in this work, water jet and UV irradiation. It has also been shown that these stability issues of ZnO can be circumvented by protecting its surface with hydrophobic coatings, which, besides conferring resistance against environmental degradation, contribute to reducing ice adhesion, yielding power efficiencies even lower than 0.5 W for ice detachment in a few seconds.

Two protective coatings were applied, CYTOP and the duplex bilayer DLC-CF_*x*_. The surface of the former did not withstand the effect of prolonged aging tests involving exposure to a water jet and UV irradiation. In contrast, the DLC-CF_*x*_ duplex coating prepared at room temperature using a one-reactor approach under mild vacuum and low power conditions, exhibited high stability and provided good acoustic wave transmission. The high elastic constant and low mass density of DLC are deemed as facilitating features for an effective surface acoustic activation of the devices. Although in their pristine state, CYTOP slightly outperformed the power efficiency for deicing at −15 °C under static conditions, the situation was reversed for IWT experiments carried out for rime and glaze ices under two angles of attack. Besides its longer durability and efficient AW transmission, another remarkable advantage of the DLC-CF_*x*_ duplex coating is its relatively low fluorine content in the thin CF_*x*_ upper layer of its structure.

The relevance of the DLC-CF_*x*_ duplex coating for SAW deicing applications is further supported by considering the deicing mechanism disclosed for the aluminum-supported SAW devices investigated in this work. Results from the icing tests, both in static and dynamic conditions, have suggested that a first deicing step consists of the SAW activation of the ice edge facing the IDT, followed by a second step involving the localized melting of a thin layer of ice interfacing with the device, specifically with the upper surface of the DLC-CF_*x*_ coating. The low thickness of this coating and, most importantly, its hydrophobic character are deemed critical factors favoring the detaching of the ice due to the low adhesion of the melted layer of water at the interface during the deicing process.

## 4 Experimental Section

### 4.1 Al\ZnO, Al\ZnO\CYTOP and Al\ZnO\DLC-CF_*x*_ Chips

SAW devices utilized in this work consisted of an aluminum substrate (1.5 mm thick), coated with a layer of piezoelectric ZnO (∼4.5 μm thickness). Two sets of IDT layouts made of Au/Cr were used for, respectively, the *Al\ZnO* and *Al\ZnO\CYTOP* chips on one side and the *Al\ZnO\DLC-CF*_*x*_ chips on the other (see Supporting Information Figure S5). The former had a 120 μm wavelength, 8 mm aperture, and 130 finger pairs. The latter had a 165 μm wavelength, 8 mm aperture and 27 effective finger pairs. The resonant frequencies of the devices were 23.3 MHz and 27.35 MHz. These IDTs presented a Single-Phase Unidirectional Transducer (SPUDT) architecture based on the classical Electrode Width Control (EWC) approach.^62^ The detailed deposition procedures and parameters used for chip manufacturing, i.e., ZnO deposition and IDT fabrication, can be found in ref 10.

### 4.2 Preparation of Coating Layers

The DLC-CF_*x*_ bilayer consisted of a DLC film of ca. 170 nm thickness and an atop CF_*x*_ film of ca. 55 nm. They were subsequently deposited by RF PECVD in a two-parallel electrode reactor using the conditions and gas precursors reported in refs 35,36 . The reactor configuration enabled the sequential deposition of layers without exposing the interfaces to air.

The CYTOP films were prepared through a solution-based process following the procedure described elsewhere^63^ from a two-part solution of CT-Solv. 180 and CTL-809 M to achieve a 1% weight of fluoropolymer chains. It is noteworthy that to improve the adhesion of the fluoropolymer chains to the ZnO surface the final steps of the fabrication procedure consisted of a three-stage baking process, first at 50 °C for 20 min, then at 80 °C for 20 min, and last, at 180 °C for 45 min.

### 4.3 Effective Power and Acoustic Wave Field Analysis

The prepared chip devices were characterized optically by digital optical microscopy (Keyence 7000, Keyence GmbH, Germany) and electrically via vector network analysis (VNA, E5080B, Keysight Technologies, USA). Due to the relatively high roughness of the aluminum substrate, the intrinsic roughness of the ZnO film, and the few imperfections or defects that may occur during manufacturing and handling processes, the electrical behavior of some device chips might be affected. Therefore, a careful evaluation of the effective power delivered to the system has been carried out. For this purpose, the S_11_ parameter measured with the VNA has been used to calculate the effective power being injected into the system. Knowing the output power of the amplifier, *P*_*amp*_, and the S_11_ of the device, the injected effective power, *P*_*eff*_, can be estimated using eq 1:

$$P_{eff}=P_{amp}\left[1-{\left[S_{11}\right]}^{2}\right]$$ 1

Throughout the article, we assess the efficiency of the deicing test by comparing the effective powers. This term encompasses both acoustic and Joule effect contributions, the latter arising from Ohmic and capacitive losses at the IDTs due to the aforementioned device imperfections.

The amplitude and phase distribution of the normal surface displacements at and around the designed frequencies of the IDTs were measured for the Al\ZnO and Al\ZnO\DLC-CF_*x*_ devices with a laser Doppler vibrometer (LDV, UHF-120, Polytec GmbH, Germany). During the measurements, the IDTs were activated with a mixed input signal composed of the same-voltage sinus signals equally spaced in the frequency domain. To suppress reflected traveling waves from the free edge in front of the IDT to the sample edge, highly viscous photoresist was added near the sample edge. Parts of the surface were sampled in a regular grid, resolving the vibrations with a resolution perpendicular to the propagation direction (Y) of 120 μm and a resolution in the propagation direction (X) of either approximately 250 μm for the overview or 1/10 of the wavelength for the detailed analysis of the deflection in front of the IDT.

### 4.4 Characterization and Functional Properties of the Coatings

Cross-section morphology of the DLC-CF_*x*_ bilayer and CYTOP films deposited on a Si wafer was characterized using a field emission scanning electron microscope (FE-SEM, Hitachi S4800). Microscale surface roughness was analyzed using an atomic force microscope (AFM, Nanotec Dulcinea microscope) operating in tapping mode. Confocal microscopy in a Sensofar metrology microscope was used to determine the long-scale distribution of roughness of the chip surfaces and the location of IDTs. Roughness parameters S_q_ and S_sk_ ^51^ were determined from optical confocal images and the S_RMS_ parameter from both optical confocal and AFM images.

Chemical analysis of the surfaces of the CF_*x*_ and CYTOP thin films was performed using XPS (SPECS) with a hemispherical analyzer (DLSEGD-Phoibos-Hsa3500) before and after exposure of the chip devices to environmental tests. The excitation source was a nonmonochromatic Al Kα radiation, and the irradiation was done in a normal configuration. Spectra were recorded with a 50 eV constant pass energy for the survey spectra and 30 eV for the zone high-resolution spectra. Binding energy (BE) was calibrated with the C 1s peak due to the C–H and C–C contributions taken at 284.5 eV in the C 1s region. A fitting analysis of the zone peaks was performed using the CasaXPS software.

The water contact angle (WCA), static and dynamic sliding angles of deionized water droplets and the freezing delay time (FDT) of frozen droplets were determined using an OCA 25 Dataphysics Instrument.^32^ The volume of the droplets was 1 μL for WCA measurements and 2 μL for FDT determination. WCA at ambient and subzero temperatures (i.e., −5.0 °C) was measured by the Young method. A minimum of five WCA measurements were made at different zones of the chip surface. The reported values are an average of these measurements and the error interval corresponds to the variation range of the measurements. FDT data were obtained in a freezing chamber with a Peltier plate following the experimental protocol reported in ref 32. Usually, the experiment was repeated three times for each sample. Data in Table 1 are the average of the obtained values. In the case of ZnO, the dispersion of values was relatively large, but always much smaller than the FDTs found for the coated chips.

### 4.5 Ice Detachment Tests

Ice detachment tests were performed using a pull-off method,^64^ whereby a motorized linear stage (IMADA MH2–500N-FA) with attached dynamometer (IMADA ZTA-200*N*/20N) applies a controlled tensile force to a threat holding an ice probe in contact with the chip surface. The probe consisted of a hollow Teflon cylinder (inner diameter 9.86 ± 0.12 mm) filled with 1 mL of Milli-Q water to a height of approximately 13 mm within the cylinder (see Figure S6). The probe was gently placed on the surface of the SAW device, approximately 2 mm from the IDT. Then, the ensemble was placed in a freezing chamber, and ice gradually formed in the probe as it cooled to a temperature of −10 °C. A constant pulling force of 4 N was applied to the probe through the thread by the dynamometer and motorized linear stage. The applied force was perpendicular to the sample’s surface (tensile mode of analysis). Under these conditions, the SAW devices were electrically excited in resonant conditions. The time required for the probe to detach was then measured and correlated with the effective power applied to the device. Data points presented some dispersion, which was larger for the Al\ZnO chips (i.e., variations of up to 10 s). As reported in Figure 5, experiments were repeated several times at very close or totally equivalent effective power values to ensure the reliability of the different tendencies found between the three investigated systems.

### 4.6 Water Jet and UV Irradiation Aging Tests

To assess the environmental stability of Al\ZnO, Al\ZnO\CYTOP, and Al\ZnO\DLC-CF_*x*_ surfaces, their resilience was tested, first, by prolonged exposure of each chip to a jet of water (e.g., simulating the effect of prolonged heavy rain), followed by the effect of UV irradiation. This way of proceeding has been considered the best option to approach the effect of the exposure of the chips to a real environment where both UV and rain will occur sequentially and, depending on conditions, simultaneously. The wetting properties of the surface were determined by comparing the water-contact angles (WCAs) measured before and immediately after the tests. Differences have been taken as evidence of possible alterations of the surface properties of samples. Accelerated water jet tests were conducted in an isothermal chamber at 2 °C with a relative humidity of 90%, exposing the chip surface to a water jet (0.04 mL s^–1^) for 3 h. Accelerated UV light irradiation tests were carried out to simulate the effect of solar radiation on the wetting properties of the examined chips. After the water jet tests, samples were exposed to the radiation produced by a 175 W ASB-XE175 Xenon light lamp equipped with an IR filter. A rough estimation of the power of the UV irradiation flux (λ < 380 nm) of this lamp at the position of the sample (15 cm) rendered a value of 0.4 W cm^–2^ (i.e., more than 1000 times higher than the irradiation intensity of the sun at ground level in this spectral region). The experiments in Figure 3 were repeated at least two times for each system, since measuring times could not be fixed at the same values for all repetitions data in this figure correspond to one of these experiments. Time evolution tendencies and final contact angles were very similar across all cases.

### 4.7 Deicing Tests of Water Droplets in Static Conditions

A water droplet (4 μL) was placed on the surface of the chip device at a distance of 10 mm from the IDTs and then brought to a freezing chamber at a temperature of −15.0 °C (Kruss Drop Shape Analyzer, DSA-30). The chips were electrically activated to generate the SAWs, and a video was recorded to monitor the melting process of the ice aggregates. Experiments were carried out with applied powers ranging from 2.5 to 5.0 W (to get effective powers within the range from 1.7 to 3.6 W for the two investigated devices). The melting process was recorded with a video camera (IDS UI-3080CP-C-HQ), and the time required to complete the melting was related to the effective power to estimate process efficiency. Experiments were conducted on pristine Al\ZnO\CYTOP, and Al\ZnO\DLC-CF_*x*_ chip devices. Each data point is the average of at least three measurements at each effective power.

### 4.8 Deicing Tests in the IWT

Deicing experiments were carried out under dynamic conditions (i.e., under the effect of an air flow) in the IWT facility located in the “Instituto Nacional de Tecnica Aeroespacial”, INTA^9,65^ with as-prepared ZnO\CYTOP and ZnO\DLC-CFx devices at two temperatures, −5.5 °C and −15.0 °C. Experiments were conducted in two steps using the experimental setup shown in Figure S9 and the following procedure: Ice was first accreted on the free surface of the devices placed at 90.0 ° with respect to the air flow direction. A collimator was used to control the shape and size of ice aggregate deposition to a defined zone on the device (see ref 10). The following ice accretion conditions were used: liquid water content (LWC) was varied from 0.14 to 0.4 g/m^3^; the medium volume diameter (MVD) of droplets was adjusted to 20 μm and the wind speed to 70 ms^–1^. These conditions and a precise control of temperature and accretion time (i.e., 1 min) were selected to induce the formation of aggregates of either compact glaze ice at −5.5 °C or rime ice at −15.0 °C, respectively. After the formation of the ice, the collimator was removed and the device tilted by either 10.0° (i.e., 100.0° with respect to the flow direction) or 45.0°. For this orientation, the infrared camera used to monitor the deicing process was perpendicular to the chip surface. This orientation also favored the upward convention of the wind, the release of heat from the surface of the device, and the eventual ejection of the interface-melted ice aggregates. Effective powers, P_eff_, of 5 W and 23 W were applied for the experiments carried out at −5.0 °C and −15 °C, respectively. The effective time, t_eff_ required to induce the slippery of the ice aggregate on the surface of the device and/or its complete removal from the chip surface has been used to estimate the energy required for the deicing (E = P_eff_t_eff_, note that slippering until detachment required maximum times of 2–3 s; occasionally a direct detachment of ice without noticeable slippering was found). Since these tests were subjected to considerable variations, each experiment was repeated at least four times. The error bar in Figure 8 reflects the large interval of variation observed for each condition, a feature supporting only a semiquantitative evaluation of the results. During the deicing process under SAW activation, videos were recorded using a video camera and a thermographic camera (Testo 890). The temperature calibration of the latter was performed by software. This calibration was referred to the initial value of the measured IR irradiance, assuming that the device and ice aggregate were at the working temperature. Changes in irradiance due to the presence of water were neglected.

## Supplementary Material















## Notes

Supporting Information includes additional physicochemical characterization and experimental details (PDF)
Video S1. Video showing the SAW-induced melting process of a small ice aggregate formed on the Al\ZnO\DLC-CF_*x*_ chip in static conditions. (MP4)
Video S2. Video showing the SAW-induced melting process of a small ice aggregate formed on the Al\ZnO\CYTOP chip in static conditions. (MP4)
Video S3. Video showing the SAW-induced interface melting and detachment process of a large ice aggregate formed on the Al\ZnO\DLC-CF_*x*_ chip. Experiment carried out in the IWT at a temperature of −5 °C. (MP4)
Video S4. Video showing the SAW-induced interface melting and detachment process of a big ice aggregate formed on the Al\ZnO\CYTOP chip. Experiment carried out in the IWT at a temperature of −5 °C. (MP4)
Video S5. Video showing the SAW-induced interface melting and detachment process of a large ice aggregate formed on the Al\ZnO\DLC-CF_*x*_ chip. Experiment carried out in the IWT at a temperature of −15 °C. (MP4)
Video S6. Video showing the SAW-induced interface melting and detachment process of a big ice aggregate formed on the Al\ZnO\CYTOP chip. Experiment carried out in the IWT at a temperature of −5 °C. (MP4)
