# Supplementary material for: Acoustic Wave-Powered Durable Icephobic Duplex Coating Design with Superior Deicing Performance
Source: ACS Appl Mater Interfaces. 2026 Jan 30;18(5):9018–31. doi: 10.1021/acsami.5c19758 (PMC12903103; doi:10.1021/acsami.5c19758)
Supplement: Supplementary file 1 [file am5c19758_si_001.pdf]

SUPPORTING INFORMATION

Acoustic wave-powered durable icephobic duplex coating design with superior de-icing performance

Jaime del Moral,[1a] Luke Haworth,[2a] Laura Montes,[1] Juan R. Sánchez-Valencia,[1] Angel Barranco,[1] Victor J. Rico,[1] Triana Czermak,[1] Francisco Carreño,[3] Paloma García-Gallego,[3] Julio Mora,[3] Carmen López-Santos,[1,4] Andreas Winkler,[5]* Ana Borrás,[1]* Agustín R. González-Elipe,[1] Yongqing Fu[2]*

1. Nanotechnology on Surfaces and Plasma Lab. Materials Science Institute of Seville. Consejo Superior de Investigaciones Científicas (CSIC)- Univ. Sevilla. Americo Vespucio 49, 41092 Sevilla, Spain
2. Faculty of Engineering and Environment, Northumbria University, Newcastle upon Tyne, NE1 8ST, UK
3. National Institute for Aerospace Technology (INTA), Ctra. Ajalvir km. 4, Torrejón de Ardoz, 28850, Spain
4. Departamento de Física Aplicada, Departamento de Física Aplicada I, Universidad de Sevilla, C/Virgen de Africa 7, Seville, 41011, Spain.
5. IFW Dresden, SAWLab Saxony, Helmholtzstr. 20, 01069 Dresden, Germany

a) Authors with equivalent contributions

E-mail: anaisabel.borras@icmse.csic.es; richard.fu@norhumbria.ac.uk; a.winkler@ifw-dresden.de

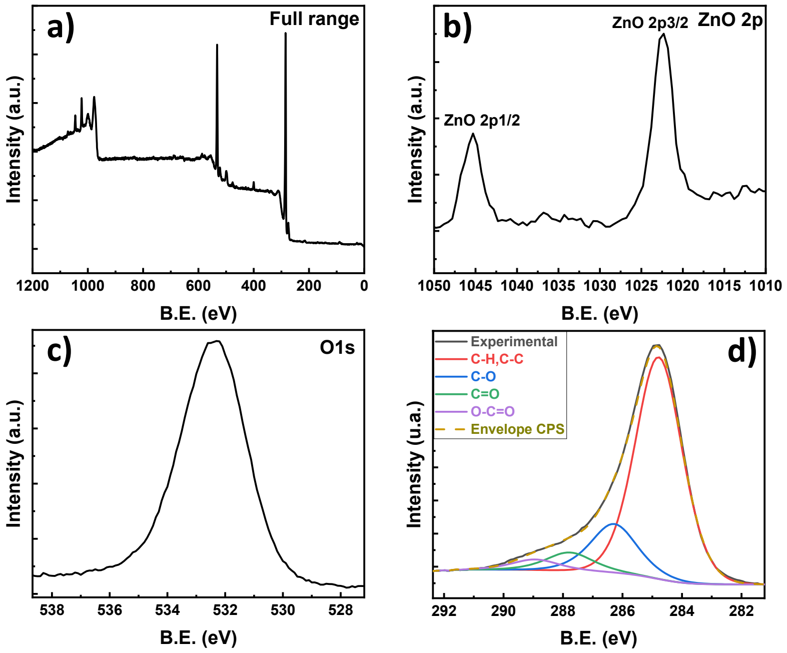

Figure S1. XPS analysis of ZnO\DLC coating. a) full range XPS spectrum, b) ZnO 2p region, c) O1s region, d) C1s region.

Table S1. Atomic composition of the ZnO\DLC coating according to XPS analysis. The first row contains the position of the photoemission peaks, and the second row shows the atomic percentage of each element.

Zn 2p	O 1s	C 1s
1022.7 eV	532.4 eV	284.7 eV
1.3 %	19.8 %	78.9 %

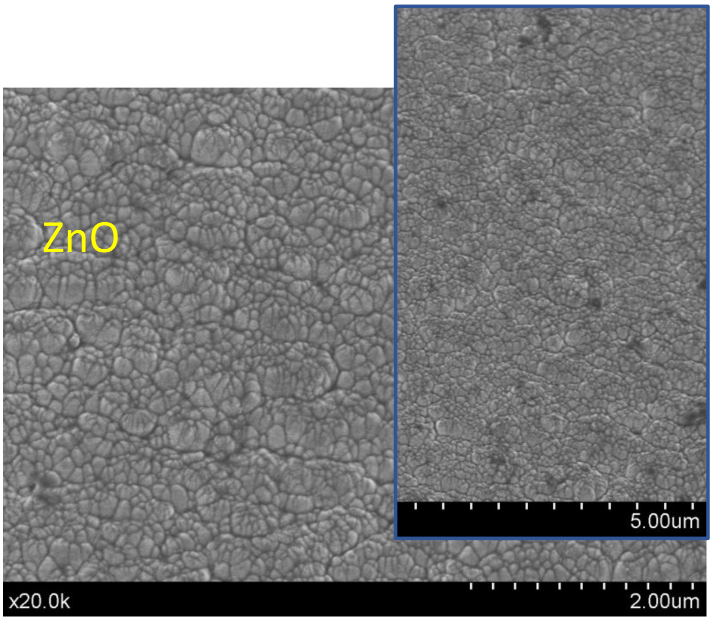

Figure S2. SEM micrographs at different magnifications of the surface state of the Al/ZnO device in its pristine state**.**

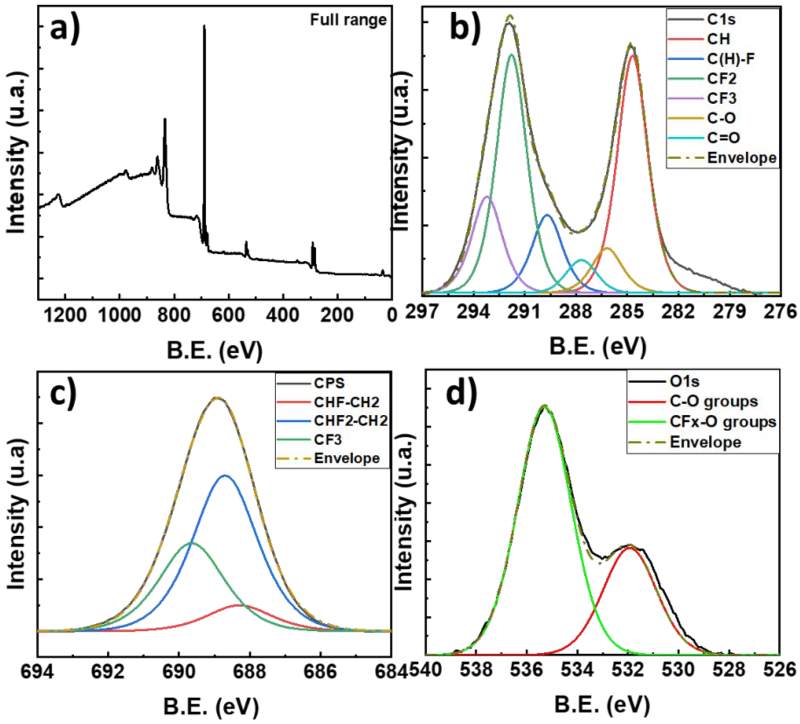

Figure S3. XPS analysis of CYTOP after aging**.** a) full range XPS spectrum, b) C1s region, c) F1s region, d) O1s region. In a-d) the black curve represents the experimental measurement, the rest of the solid lines represent the fitting used in the chemical analysis, and the dashed lines in dark yellow represent the final fitting.

Table S2. Chemical composition of the CYTOP coating according to XPS analysis. The first row contains the position of the photoemission peaks, and the second row shows the atomic percentage of each element relative to the total surface composition.

F1s					
**CHF-CH2-**		**CHF2-CH2-**		**-CF3**	
688.30 eV		688.7 eV		689.6 eV	
5.0 %		30.2 %		17.0 %	
**O1s**					
**O-C***			**O-CFx***		
531.9 eV			535.3 eV		
1.5 %			3.59 %		
**C1s**					
**-C-**	**-CF-**	**-CF_2-**	**-CF_3-**	**-C-O**	**-C=O**
284.80 eV	289.70 eV	291.78 eV	293.22 eV	286.19 eV	287.69 eV
13.9 %	4.6 %	14.0%	5.6 %	2.6 %	1.83 %

** These are not specific species but families of functional groups*

Supporting information S4. Roughness assessment of the surface of chip devices subjected to the aging treatments.

Figure S5 presents some confocal images taken of the three chips after their exposure to the aging tests described in the main text. Table S5 shows data corresponding to the roughness parameters Sq and Ssk determined for the three samples from the analysis of these images.

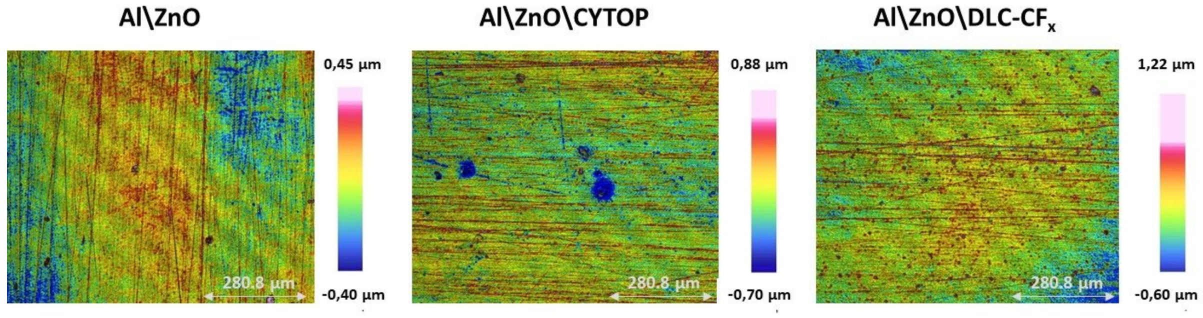

Figure S4. Confocal images taken for the chips subjected to the aging treatments (see main text)

Table S3. Roughness parameters determined from the images in Figure S4.

Roughness parameter	Pristine Al\ZnO	Aged Al\ZnO	Aged Al\ZnO\CYTOP	Aged Al\ZnO\DLC-CF_x
**Sq**	0.37 µm	0.13 µm	0.21 µm	0.24 µm
**Ssk**	8.12	-0.07	0.41	1.09

A method for estimating the flatness and proportion of hills and valleys on rough surfaces is through the so-called SsK parameter (a definition of this parameter can be found in ref. S1). With respect to an ideal plane defined on the surface, SsK<0 values indicate a significant contribution of valleys, Ssk values equal to or close to zero indicate a compensated proportion of valleys and hilltops (or a flat topography), while Ssk>0 indicates a preponderance of hilltops.

Supporting information S5.- IDT layout of Al\ZnO and Al\ZnO\DLC-CF_x devices

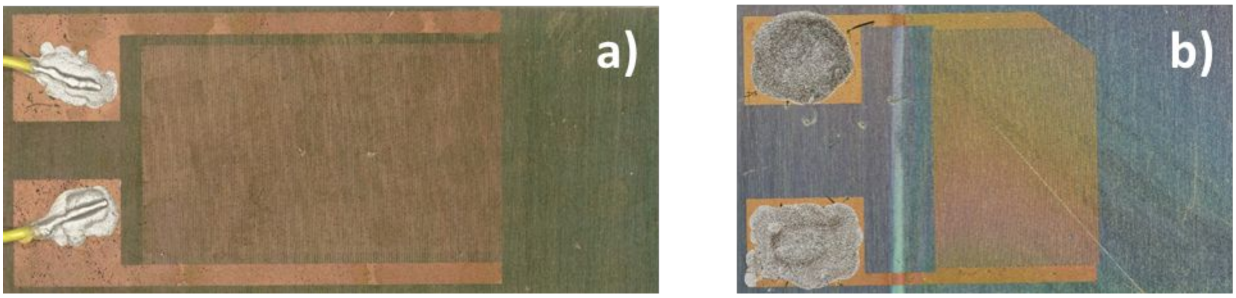

Figure S5. Digital optical micrograph of the analysed IDTs: a) Al\ZnO and Al\ZnO\CYTOP and b) Al\ZnO\DLC-CF_x. The details of the device layouts are the following:

- Al\ZnO: Unidirectional IDT layout with 120µm wavelength, 8mm aperture, and 130 Finger pairs.
- Al\ZnO\DLC-CF_x: Bidirectional $\lambda/4$ IDT layout, 165µm wavelength, 8mm aperture and 35 Finger pairs (whereby 8 fingers on one side of the IDT were not electrically connected to the bus bar)

Experimental setup used for the ice detachment tests

The ice-detachment experiments were conducted using the setup illustrated in Figure S6. In a cold chamber maintained at -10 ºC, the sample is secured with screws onto a steel plate, which provides a stable support. A nylon cylinder with a specified cross-section is positioned on the surface, 2 mm away from the end of the interdigital transducers (IDTs). The cylinder is then partially filled with 1 mL of Milli-Q water. Once the water freezes, the ice adheres to both the walls of the cylinder and the surface of the sample, exhibiting a certain ice-adhesion strength. The top of the cylinder is connected to a cable that leads to a dynamometer at the opposite end. The dynamometer is mounted on a motorized linear stage. This setup allows for the application of a fixed tensile tension (τ) to the ice by pulling the cable. In the experiments conducted, this tensile tension was maintained at 52.38 KPa (equivalent to 4N applied in the dynamometer).

In contrast to ice-adhesion experiments, ice-detachment experiments focus on measuring the duration for which the ice-sample interface can withstand a specific tensile tension and acoustic power. The experiments

herein consisted of varying the total applied power from 4 W to 13 W. The effective power injected into each sample was determined using the S11 parameter. Each power level was tested at least three times, and the average of these repetitions was taken as the final value for analysis.

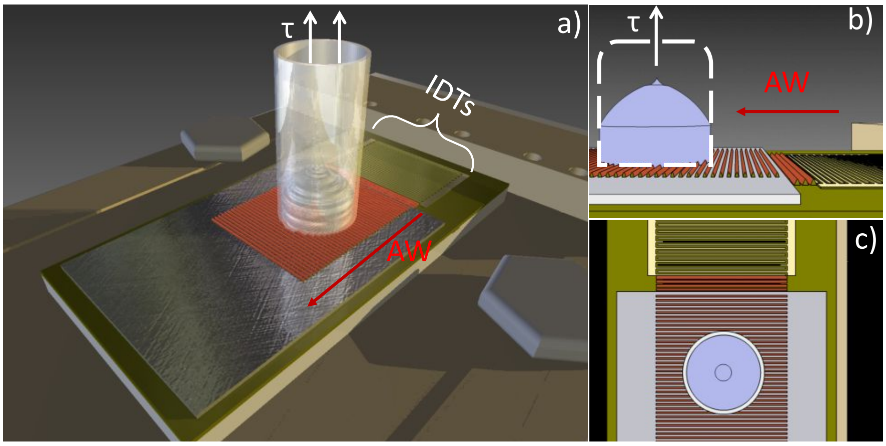

Figure S6. Experimental set-up of Ice-detachment experiments. Sketch of the experimental setup from different perspectives with a visualization of the acoustic waves (red curves).

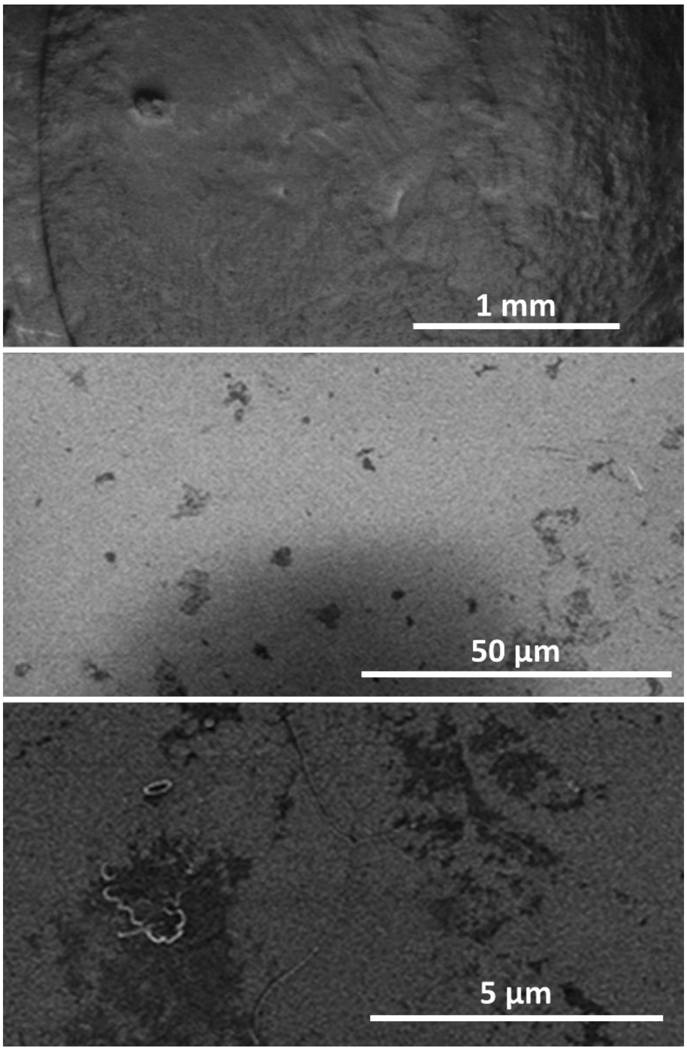

Figure S7. SEM images at different magnifications taken for the Al\ZnO\CYTOP chip in the zone where the ice probe was located.

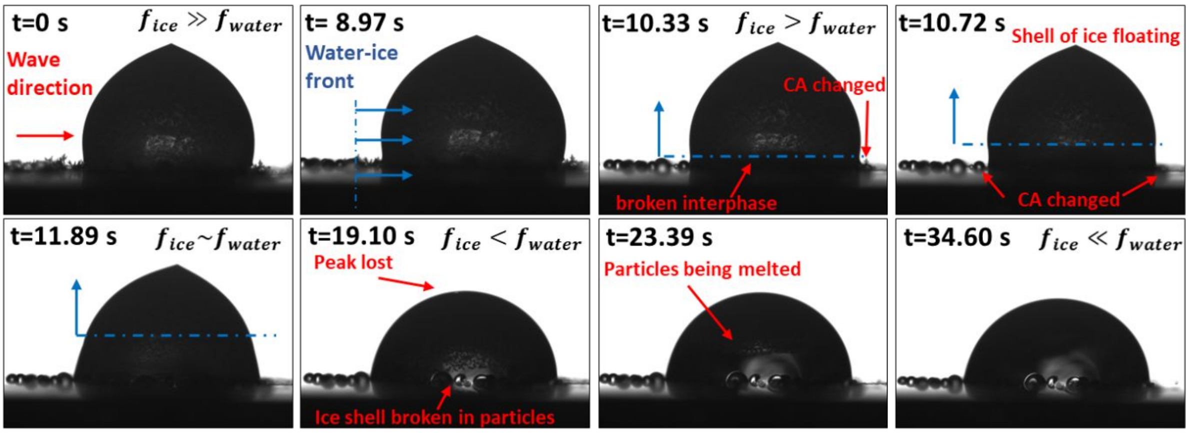

Figure S8. Snapshots of a small glaze ice aggregate formed under static conditions during activation of an Al\ZnO\CYTOP chip at an effective applied power of 3.56 W.

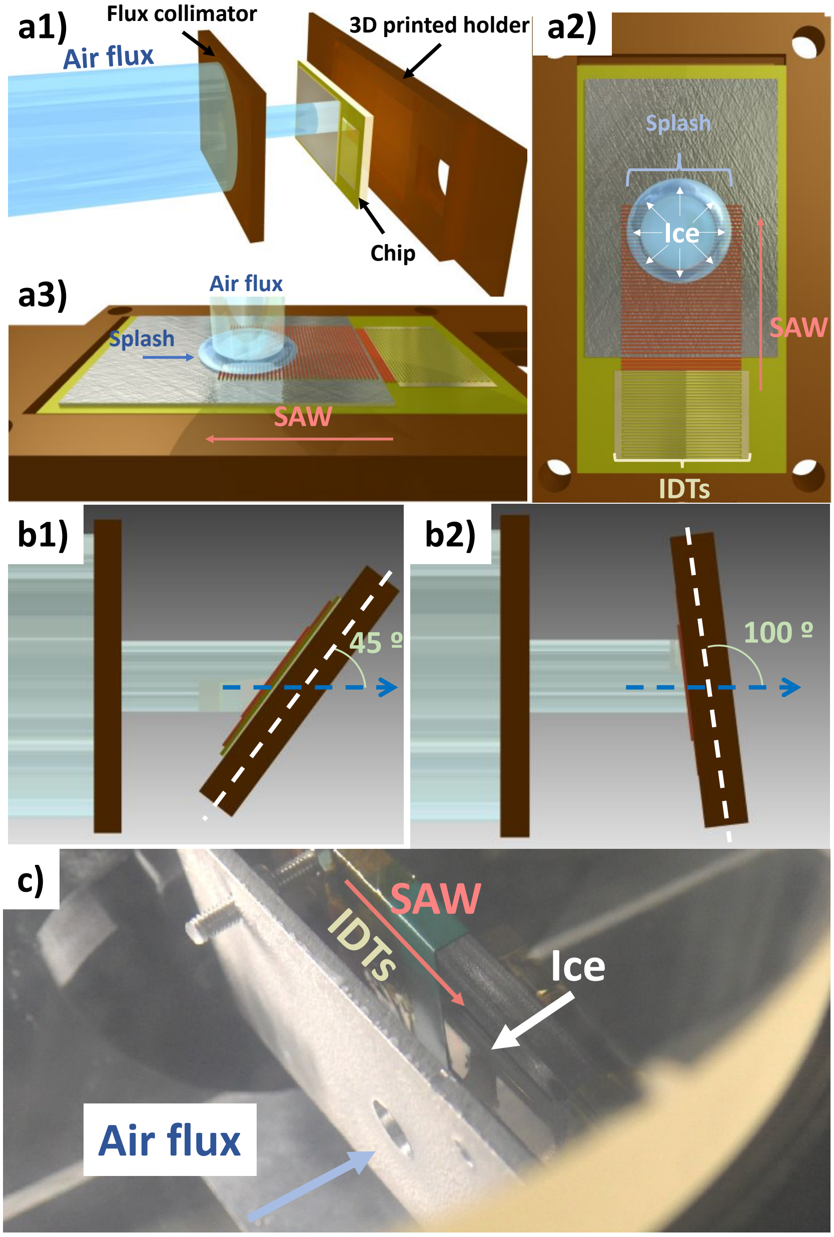

Figure S9. Experimental set-up in IWT. a1-3) diagram of the set-up in INTA from different angles, b1-2) schematic of the two angles of exposition to the wind; c) picture of the device under experimentation at the IWT.

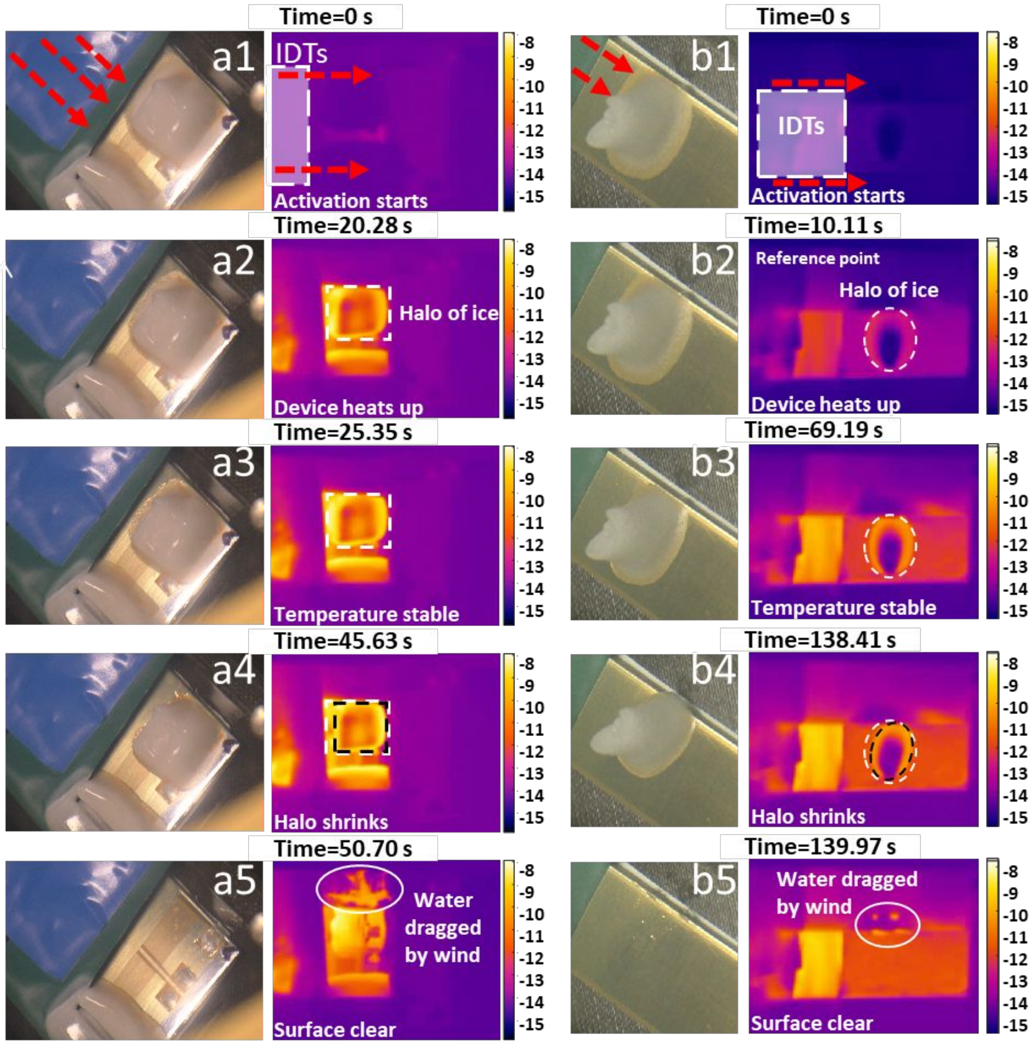

Figure S10. SAW induced de-icing of large ice aggregates in the IWT for the Al\ZnO\DLC-CF_x (a) and Al-ZnO-CYTOP (b) at -15 ºC for the devices placed at an angle of 45º with respect to the wind flow.

References

S1. Horcas, I.; Fernández, R.; Gómez-Rodríguez, J. M.; Colchero, J.; Gómez-Herrero, J.; Baro, A. M. WSXM: A Software for Scanning Probe Microscopy and a Tool for Nanotechnology. Rev. Sci. Instrum. 2007, 78 (1), 013705. https://doi.org/10.1063/1.2432410.

Supporting video descriptions

Video S1.-Video showing the SAW-induced melting process of a small ice aggregate formed on the Al\ZnO\DLC-CF_x chip in static conditions.

Video S2.-Video showing the SAW-induced melting process of a small ice aggregate formed on the Al\ZnO\CYTOP chip in static conditions.

Video S3.-Video showing the SAW-induced interface melting and detachment process of a big ice aggregate formed on the Al\ZnO\DLC-CF_x chip. Experiment carried out in the IWT at a temperature of -5ºC.

Video S4.- Video showing the SAW-induced interface melting and detachment process of a big ice aggregate formed on the Al\ZnO\CYTOP chip. Experiment carried out in the IWT at a temperature of -5ºC.

Video S5.- Video showing the SAW-induced interface melting and detachment process of a big ice aggregate formed on the Al\ZnO\DLC-CF_x chip. Experiment carried out in the IWT at a temperature of -15ºC.

Video S6.- Video showing the SAW-induced interface melting and detachment process of a big ice aggregate formed on the Al\ZnO\CYTOP chip. Experiment carried out in the IWT at a temperature of -5ºC.
